# Metabolomics and Physiological Insights into the Ability of Exogenously Applied Chlorogenic Acid and Hesperidin to Modulate Salt Stress in Lettuce Distinctively

**DOI:** 10.3390/molecules26206291

**Published:** 2021-10-18

**Authors:** Leilei Zhang, Begoña Miras-Moreno, Evren Yildiztugay, Ceyda Ozfidan-Konakci, Busra Arikan, Fevzi Elbasan, Gunes Ak, Youssef Rouphael, Gokhan Zengin, Luigi Lucini

**Affiliations:** 1 Department for Sustainable Food Process, Università Cattolica del Sacro Cuore, Via Emilia Parmense 84, 29122 Piacenza, Italy; leilei.zhang@unicatt.it (L.Z.); luigi.lucini@unicatt.it (L.L.); 2 Department of Biotechnology, Faculty of Science, Selcuk University, Selcuklu, Konya 42130, Turkey; eytugay@selcuk.edu.tr (E.Y.); busra.arikan@selcuk.edu.tr (B.A.); fevzi.elba@gmail.com (F.E.); 3 Department of Molecular Biology and Genetics, Faculty of Science, Necmettin Erbakan University, Meram, Konya 42090, Turkey; cozfidan@erbakan.edu.tr; 4 Physiology and Biochemistry Research Laboratory, Department of Biology, Science Faculty, Selcuk University, Selcuklu, Konya 42130, Turkey; akguneselcuk@gmail.com (G.A.); gokhanzengin@selcuk.edu.tr (G.Z.); 5 Department of Agriculture, University of Naples Federico II, Via Università 100, 80055 Portici, Italy

**Keywords:** metabolomics, salinity, plant stress, redox imbalance, stress alleviator

## Abstract

Recent studies in the agronomic field indicate that the exogenous application of polyphenols can provide tolerance against various stresses in plants. However, the molecular processes underlying stress mitigation remain unclear, and little is known about the impact of exogenously applied phenolics, especially in combination with salinity. In this work, the impacts of exogenously applied chlorogenic acid (CA), hesperidin (HES), and their combination (HES + CA) have been investigated in lettuce (*Lactuca sativa* L.) through untargeted metabolomics to evaluate mitigation effects against salinity. Growth parameters, physiological measurements, leaf relative water content, and osmotic potential as well as gas exchange parameters were also measured. As expected, salinity produced a significant decline in the physiological and biochemical parameters of lettuce. However, the treatments with exogenous phenolics, particularly HES and HES + CA, allowed lettuce to cope with salt stress condition. Interestingly, the treatments triggered a broad metabolic reprogramming that involved secondary metabolism and small molecules such as electron carriers, enzyme cofactors, and vitamins. Under salinity conditions, CA and HES + CA distinctively elicited secondary metabolism, nitrogen-containing compounds, osmoprotectants, and polyamines.

## 1. Introduction

With the continuous expansion of the population, the importance of development studies on natural resources and agriculture is increasing day by day, while global warming and environmental pollution lead to the limitation of agricultural activities [1]. Exposure to abiotic stresses, including salinity, heat, drought, flooding, and heavy metal toxicity, prevents plants from reaching their full growth potential and reduces many crops’ yield [2]. Soil and water salinity is currently one of the major problems, and it is affecting more than nearly 20% of the world’s irrigated land at different levels [3]. Most of the adverse effects of salinity are due to the increase and accumulation of Na^+^ and Cl^−^ ions in plants, which is called ionic stress. The interaction of these ions with critical metabolic processes causes irreversible changes such as pigment degradation, disruption of enzyme and protein structures, and photosynthesis inhibition. In addition, osmotic stress caused by these ions disrupts the balance of water and nutrient uptake in the roots, causing loss of cellular homeostasis [4]. Osmotic stress triggers damage to the membranes of mitochondria and chloroplasts, disrupting photosynthesis and energy production processes. Indeed, some authors reported that the regulation of chlorophyll biosynthesis in plants depends on the type of stress applied, producing important consequences on the growth and reduction of biomass [5,6,7]. To overcome salinity-mediated growth reduction, plants activate tolerance mechanisms such as antioxidant systems, osmolyte accumulation, and the compartmentalization of toxic ions [8]. Many studies have previously shown that the production and accumulation of reactive oxygen species (ROS) such as superoxide (O_2_^−^), hydrogen peroxide (H_2_O_2_), and singlet oxygen are parallel with exposure time and density in plants exposed to salt stress. Accumulated ROSs cause damages to membrane integrity by initiating the lipid peroxidation process [9]. Plants have developed enzymatic and non-enzymatic antioxidant systems to protect them from the destructive effects of oxidative stress [10].

Recent studies in the agronomic field focus on more sustainable alternatives that can be used against abiotic stresses and yield loss in plants. Studies have also shown that polyphenols provide tolerance to the plant against various stresses by exogenous application [11,12,13]. These biologically active molecules can play different metabolic roles according to their structural differences and are an essential part of plants’ stress response [14]. For example, vanillic acid application to tomato seedlings under salinity improved chlorophyll synthesis and plant growth by reducing osmotic and oxidative stress [15]. Similarly, Mekawy et al. [16] showed that apigenin treatment enhances tolerance to salinity stress by supporting the antioxidant system in rice plants. These polyphenols with high ROS scavenging capacity as hydrogen donors provide defense against abiotic stress in plants by supporting the antioxidant system [14].

Plant polyphenols show a large chemical diversity, to include phenolic acids, stilbenoids, flavonoids, and lignans, synthesized through the phenylpropanoid pathway [17]. Chlorogenic acid (CA), a phenolic acid, and hesperidin (HES) from the group of flavonoids are bioactive molecules whose health-promoting, antimicrobial, and antiviral properties have been shown in many studies [18,19]. Nonetheless, CA also participates in the development and growth of plants [20]. Mei et al. [21] showed that exogenous CA reversed membrane damage and lipid oxidation and induced the transcription of genes related to antioxidant capacity, including peroxidase, catalase, and polyphenol oxidase. The reports about the antioxidant properties of HES have been mainly focused on animals [22,23]. In planta, [12] demonstrated that HES accumulation increased in *Cyclopia subternata* callus under UV light. However, there are no further data on the impacts of exogenously applied HES on plant biochemistry in response to stress. Based on previous information, the application of these flavonoids to plants may provide protective effects against stressors. Moreover, plants exposed to combined CA and HES can provide complementary and synergic effects. Indeed, our previous work [24] showed that CA, HES, and their combination modulated the functional traits of lettuce in a distinctive manner.

Lettuce (*Lactuca sativa* L.), categorized as relatively salt-sensitive, showed impaired germination, membrane permeability, redox balance, and photosynthetic performance under salinity [25,26,27]. Therefore, this crop represents a good model to study the effects of salinity. Most of the previous studies focused on growth, photosynthetic capacity, nutritional parameters, and crop quality in stress-treated lettuce plants [28,29,30,31]. Based on our previous information on the ability of phenolics to modulate the functional traits in lettuce, the present work aimed at investigating the effect of CA, HES, and their combination on lettuce under salt stress conditions, compared to non-stressed lettuce plants. To this object, a combination of physiological measurements and untargeted metabolomics has been used to unravel lettuce changes at the biochemical level, with a focus on stress mitigation effects.

## 2. Results

### 2.1. Plant Growth and Ecophysiological Changes

**Figure 1:**
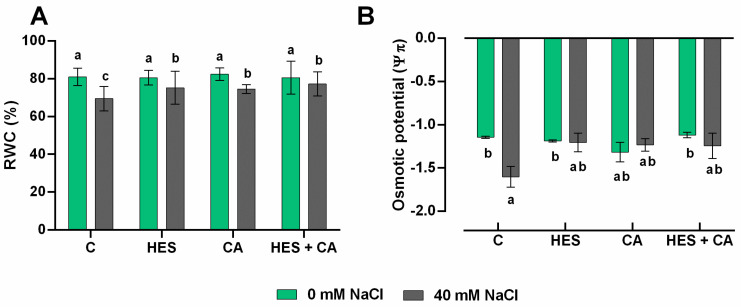
(**A**) Relative water content (RWC) and (**B**) leaf osmotic potential (Ψπ) of lettuce after 10 days of treatments, the values are expressed as mean percentage ± standard deviation of four independent replicates. The lettuce plants were treated with: control no treated (C), 100 μM hesperidin (HES), 50 μM chlorogenic acid (CA), 100 μM HES + 50 μM CA. Treatments were performed under both salt (40 mM NaCl) and non-salt (0 mM NaCl) stress. The statistical significance has been tested by a one-way ANOVA test with Tukey post-test multiple comparisons (*p*-value < 0.05), and significant differences between treatments are indicated by lowercase letters above each bar.

Lettuce shoot fresh and dry weight were not significantly affected by salinity or phenolics (either alone or in combination) (Supplementary Materials Figure S1). However, the plant relative water content (RWC) significantly decreased in NaCl-treated plants compared to the control plants (Figure 1A). Interestingly, this detrimental effect was not observed when exogenous HES and HES + CA (*p* < 0.05) were applied under salinity. However, a slight enhancement of RWC was observed for CA treatment (Figure 1A), whereas the exogenous application of phenolics did not affect RWC in non-stressed plants. On the other hand, leaf osmotic potential (Ψπ) was significantly affected by salinity, with an increase of 27%, compared to the non-stressed control (Figure 1B). The treatment with exogenous phenolics did not present differences compared to both the control and the NaCl treatment, suggesting a non-significant mitigation for this parameter.

**Figure 2:**
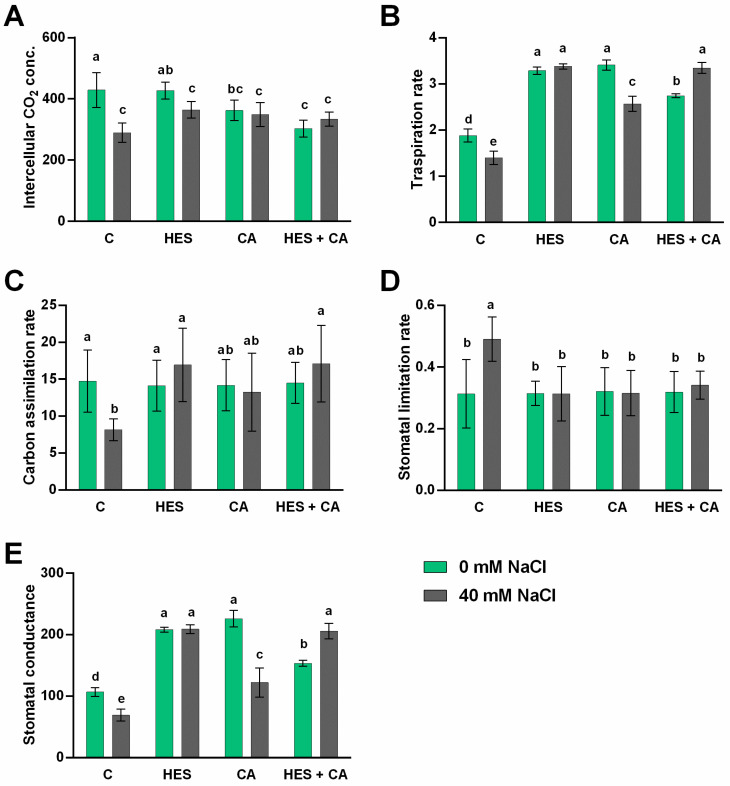
(**A**) Intercellular CO_2_ concentration (Ci), (**B**) transpiration rate (E), (**C**) carbon assimilation rate (An), (**D**) stomatal limitation rate (Ls), and (**E**) stomatal conductance (gs) of lettuce after 10 days of treatments; the values are expressed as mean percentage ± standard deviation of eight replicates. The lettuce plants were treated with: control no treated (**C**), 100 μM hesperidin (HES), 50 μM chlorogenic acid (CA), 100 μM HES + 50 μM CA. Treatments were performed both under salt (40 mM NaCl) and non-salt (0 mM NaCl) stresses. The statistical significance has been tested by a one-way ANOVA test with Tukey post-test multiple comparisons (*p*-value < 0.05), and significant differences between treatments are indicated by the lowercase letters above each bar.

The leaf gas exchange parameters, such as intercellular CO_2_ concentration (Ci), carbon assimilation rate (An), transpiration rate (E), stomatal limitation rate (Ls), and stomatal conductance (gs) were also investigated. Leaf gas exchange was negatively affected by salt stress (*p*-value < 0.05; Figure 2A–E). Nevertheless, as in the case of RWC, the exogenous application of HES or HES combined with CA effectively mitigated the negative effect of salt stress on gas exchange parameters. Indeed, regarding the stomatal limitation rate (Ls), salinity stress reduced Ls by about 34% compared to the control. At the same time, both HES and CA were able to recover the Ls rate into a physiological state (Figure 2D). Similarly, the stomatal conductance (gs) and transpiration rate (E) of lettuces subjected to stress were significantly affected by the treatments. In this case, phenolics were able to enhance both gs and E values by two-fold compared to both stressed and non-stressed controls. Interestingly, HES-treated lettuces strongly enhanced gs and E parameters (i.e., 205 mol m^−2^ s^−1^ and 3.38 mmol H_2_O m^−2^ s^−1^, respectively), particularly under stress conditions (Figure 2B,E).

The same trend was observed for carbon assimilation rate (An), which was highly affected by salinity (An < 8 μmol m^−2^ s^−1^). The treatments with exogenous phenolics led to a significant increase in An (16–17 μmol m^−2^ s^−1^), revealing a higher capacity to assimilate C than control plants (13–15 μmol m^−2^ s^−1^), as shown in Figure 2C. On the other hand, the intracellular CO_2_ concentration (Ci) decreased by about 25% in NaCl-treated plants, and only HES-treated lettuce presented a significant recovery of Ci (Figure 2A).

### 2.2. Metabolomics

**Figure 3:**
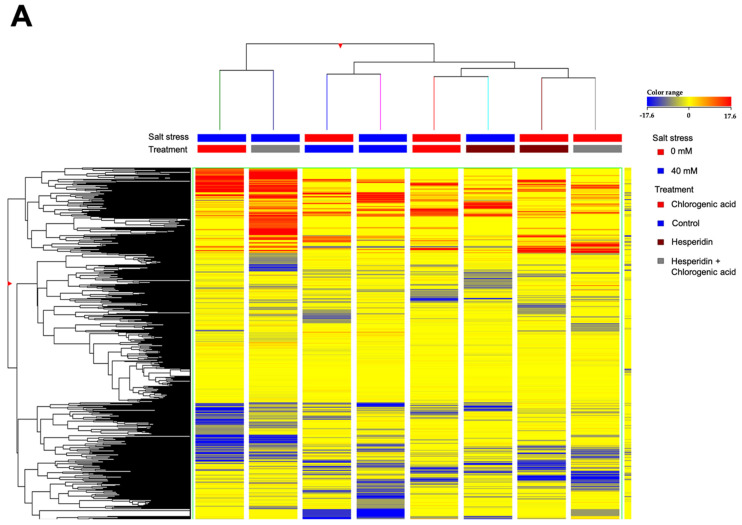

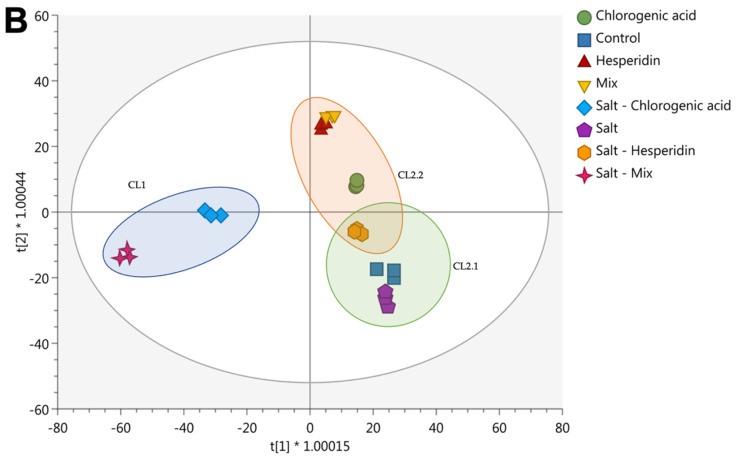
(**A**) Unsupervised hierarchical cluster analysis (HCA) obtained from fold-change values and (**B**) Orthogonal projection to latent structures discriminant analysis (OPLS-DA) supervised modeling of lettuce metabolome after 10 days of treatments. The lettuce vegetables were treated with: control no treated, 100 μM hesperidin (HES), 50 μM chlorogenic acid (CA), 100 μM HES + 50 μM CA (Mix). Treatments were performed both under salt (40 mM NaCl) and no-salt (0 mM NaCl) stress. *: mathematical multiplication.

An untargeted metabolomics approach was adopted to investigate the differences in metabolic responses of lettuces exposed to salt stress treated with HES, CA, and HES + CA. This approach allowed us to putatively annotate more than 3700 compounds, and the comprehensive list of annotations is provided in the supplementary material together with individual abundances and composite mass spectra. The unsupervised hierarchical cluster analysis (HCA), made from a fold-change heatmap built on values normalized by the median, was carried out to group different treatments according to their similarity/dissimilarity in metabolic profiles (Figure 3A). The HCA produced two main clusters; the first cluster included treatments with CA and HES + CA grown under salinity, while the second cluster was in turn composed of two different sub-clusters, i.e., controlled salinity and non-salinity, and treatments with CA, HES, and HES + CA under non-salinity and HES under salinity. The clustering results suggest that lettuces treated with CA and HES + CA grown under salinity showed a similar metabolic profile and that treatments with both CA and HES (alone and combined) under non-stressed conditions display distinctive metabolic profiles closer to the controls.

After that, a supervised OPLS discriminant multivariate analysis was made to model metabolomic signatures and confirm the output obtained by the unsupervised model (Figure 3B). The goodness of prediction and goodness of fit parameters of the model were Q^2^Y = 0.773 and R^2^Y = 0.986, respectively. In addition, the model was cross-validated (*p*-value = 2.82 × 10^−8^), inspected for outliers (by Hotelling’s Test), and model overfitting (by permutation testing; *n* = 100) (Figure S2). The model parameters suggest the high robustness and accuracy of the model. Regarding the output, the OPLS score plot confirmed patterns from HCA, and different clusters obtained are grouped (Figure 3B). Specifically, the first component of the model t[1] discriminates the CL1 from the CL2, confirming a distinctive metabolic profile of lettuces treated with CA and HES + CA under salinity stress. The second component t[2] showed the ability to clarify the differences within the CL2. Compared to HCA, the OPLS model suggests that the HES-treated lettuce grown under salinity stress had results closer to the controls than the other treatments. Overall, the HES + CA treatment showed a different behavior depending on the presence or absence of stress. Indeed, in the presence of salinity stress, the metabolic profile was similar to that found with the CA treatment, while in the absence of stress, it is similar to the profile obtained from treatment with HES.

Finally, 47 VIP markers were selected (by setting the VIP score threshold to >1.3; Supplementary Tables), representing the compounds with the highest discrimination potential. Overall, these VIP compounds were involved in secondary metabolism, including several cofactors, and fatty acid and lipid biosynthesis. Among secondary metabolites, the most discriminant VIP compounds were alkaloids, phenylpropanoids, terpenoids, and sesquiterpenes. Moreover, compounds related to chlorophyll, phytohormones, and polyamines biosynthesis have also been detected.

#### 2.2.1. Effect of the Treatments on Plant Metabolomic Profiles under Non-Stress Conditions

**Figure 4:**
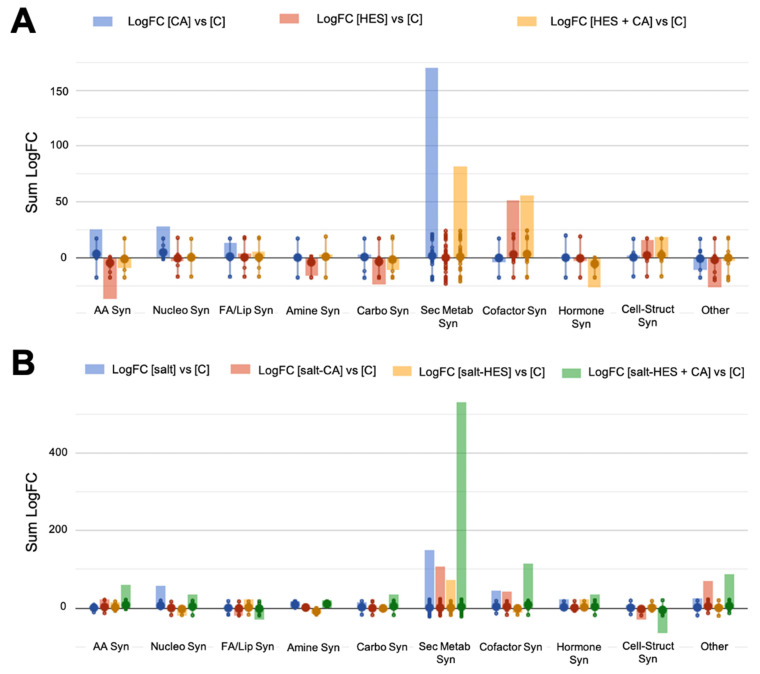
Biosynthesis pathways carried out from lettuce vegetables after 10-day treatments with 100 μM hesperidin (HES), 50 μM chlorogenic acid (CA), and 100 μM HES + 50 μM CA (Mix), grown under non-stressed condition (**A**) and salt-stressed condition (**B**). The dataset used to carry out the metabolomic analysis was produced through UHPLC-ESI/QTOF-MS and followed by a selection of compounds that pass ANOVA and FC analysis (*p*-value < 0.05, fold-change > 1.3), and differential metabolites were loaded into the PlantCyc Pathway Tool (https://www.plantcyc.org/, accessed on 1 July 2021). The abbreviated subcategory names on the x-axis correspond to: AA: amino acids; Nucleo: nucleosides and nucleotides; FA/Lipids: fatty acids and lipids; Amines: amines and polyamines; Carbo: carbohydrates; Cofactors: cofactors, prosthetic groups, electron carriers, and vitamins; Cell-Struct: Cell structures; Syn: Synthesis.

The effect of CA and HES treatments on lettuce grown under non-stress conditions was investigated to unravel the modulation of metabolism after its application. As suggested by multivariate statistics, the exogenous application of phenolics largely modified the metabolic profile of lettuce plants, with 256 metabolites significantly affected by the treatment, compared to control. These distinctive compounds were interpreted using the online tool Omic Dashboard from PlantCyc. A complete list of compounds passing ANOVA and FC is provided in the Supplementary Tables. The output of the Omic Dashboard is reported in Figure 4A, and the summary table of pathways involved in response to different treatments is provided in Table 1.

Overall, the different treatments produced a relevant stimulation of secondary metabolism in lettuces, with 113 significant compounds involved in this pathway, followed by cofactors, carriers, and vitamins biosynthesis, and fatty acid and lipid biosynthesis (Table 1). However, the sole presence of CA or HES implied a distinct metabolic response compared to the control and compared to the combined treatment, suggesting that the combined application triggered a distinguished plant response from the individual treatment. Lettuce treated with CA and HES + CA resulted in an up-accumulation of secondary metabolites by 170.39 and 82.39 cumulative LogFC value, respectively (Figure 4A and Table 1), while the treatment with HES did not show a clear trend of accumulation compared to the control. In particular, all the treatments broadly elicited terpenes, phytoalexins, and nitrogen-containing secondary metabolites. Interestingly, also phenylpropanoids derivatives (hydroxyflavones, isoflavones, and coumarins) were up-accumulated after CA treatment (Supplementary Figure S3A).

However, although HES and HES + CA application resulted in a general down-accumulation of phenylpropanoids, a remarkable accumulation of flavonoids (hydroxyflavones, flavonols, and anthocyanins), lignans, and coumarins could be observed. The biosynthesis of cofactors, electron carriers, and vitamins that participate in a variety of enzyme reactions were accumulated after HES and HES + CA application. Among others, intermediates involved in folate and flavin biosynthesis, and pheophorbide, increased. Regarding the fatty acid and lipid biosynthesis pathway, the exogenous application of phenolics produced an up-accumulation of 13.67, 4.29, and 5.72 sum LogFC values for CA, HES, and HES + CA, respectively (Table 1). The classes of lipids involved were sterols, phospholipids, and fatty acids. Interestingly, all three treatments positively modulated the choline-related pathway. Polyamines, in particular, compounds related to spermine and spermidine, were also elicited by the treatments. Finally, the precursor of proline, 1-pyrroline-5-carboxylate, was strongly accumulated in CA- and HES + CA-treated plants.

#### 2.2.2. Effect of the Treatments on Metabolomic Profiles under Stress Conditions

Once the effect of exogenous phenolic was determined, the differential plant response to the exogenous application of phenolics under stress conditions was investigated on lettuces using the same method previously described for lettuces treated under non-stressed conditions. The combined ANOVA and FC analysis resulted in 429 discriminant metabolites, suggesting a higher effect of phenolic compounds under adverse conditions (Supplementary Tables). This differential modulation of plant response by the exogenous phenolics under stress conditions, suggested by the multivariate statistics, was confirmed by ANOVA and FC, since most of the compounds found as discriminant were found in the aggregated presence of salt and phenolics (Figure 4B). The summary table of pathways involved in plant response to different treatments is reported in Table 2.

In particular, 40 mM NaCl triggered the plant defense response with a positive modulation of plant secondary metabolism (Table 2). Together with the previously mentioned increase in cofactors, electron carriers, vitamins, and polyamines, the synthesis of macromolecules such as cell structure components, fatty acids, and lipids was also observed (Figure 4B). The multivariate analysis suggests the greater effect of the combined treatment (HES + CA), indicating a synergistic effect. In detail, the combined treatment with HES and CA induced a significant accumulation of secondary metabolites (Table 2) compared to the control. The treatment with CA showed a similar trend of metabolite accumulation; its metabolic profile recovered more than that of the salinity-treated control sample. At the same time, the HES application resulted in metabolomic signatures comparable to control plants, differently from other treatments. The main pathways elicited by this treatment involved terpenoids, nitrogen- and sulfur-containing compounds, phenylpropanoids, and phytoalexins. These rises were even more evident for HES + CA treatment. Concerning phenylpropanoid, an accumulation of precursors required for their biosynthesis, such as phenolic malonylglucosides and l-phenylalanine, was observed. In addition, the results pointed out a marked modulation of glucosinolates by all phenolic treatments under stress conditions (Supplementary Figure S4). Moreover, an up-accumulation of electron carriers, enzyme cofactors, and vitamins after CA and HES + CA treatments could be recorded, whereas an opposite trend was observed in samples treated with HES.

On the other hand, stress-related hormones were confirmed to be modulated by NaCl as well as by the exogenous application of phenolics. 7′-hydroxyabscisate was strongly accumulated in HES- and HES + CA-treated plants under non-stress conditions and in CA- and HES + CA-treated plants under stress conditions. Jasmonates were also modulated mainly in the presence of HES, regardless of the presence of the stress or not. However, brassinosteroids accumulation was promoted in the presence of NaCl and reverted back by phenolics.

Finally, osmolytes were also involved in plant response to phenolics under salinity. Pyrroline-5-carboxylate, hydroxy-5-oxoproline, and oxo-L-proline (all proline-related metabolites) were strongly accumulated in plants treated mainly with CA and HES + CA, while betaine aldehyde was accumulated in NaCl- and HES + CA-treated plants. 

## 3. Discussion

Abiotic stresses, including UV radiation, heat, cold, salinity, drought, and heavy metals, dramatically impact plant development by affecting several biochemical and physiological processes such as photosynthesis, hormonal signaling, and antioxidant systems, including the generation and homeostasis of reactive oxygen species (ROS), leading to a cellular chemical imbalance [32]. In this sense, several studies reported that polyphenols provide tolerance to the plant against various stresses by exogenous application [11,12,13]. However, little is known about the biochemical mechanisms implied in the plant response to the application of exogenous phenolics. In this study, we investigated the effect of two phenolic compounds, HES and CA, exogenously applied on the ecophysiological parameters and on the metabolic profiles of lettuces exposed to moderate salt stress (40 mM NaCl) to unravel the plant response triggered by these phenolics, either alone or in combination.

Regarding the physiological parameters, the damage produced by salinity was confirmed at the level of stomatal capacity and stomatal conductance (Figure 2D,E). However, both CA and HES and their combination (HES + CA) mitigated the negative effect of salt stress (RWC, E, An, Ls, and gs). No differences were observed regarding biomass, not even under stress conditions, which is probably due to the moderate stress applied (40 mM NaCl for 10 days). RWC is a realistic measure of plant water status consequently to a condition of stress. This parameter indicates the capacity of the plant to transport water from the soil to atmosphere. The osmotic potential is a mechanism activated by cells to conserve hydration when subjected to stress such as salinity, and interestingly, the treatments with phenolics allowed recovering the physiological statement of both relative water content and leaf osmotic potential. In this regard, when plants are subjected to salinity, stomatal guard cells limit stomatal conductance to reduce the transpiration rate, consequently impairing the photosynthesis and gas exchange parameters [33] (Figure 2). Indeed, the photosynthesis system is reported as one of the first physiological processes affected by saline stress, and Qin et al. [8] reported a direct correlation between the amelioration of gas exchange parameters, affected by salinity, and the recovery of photosynthesis activity, after the exogenous application of acetylcholine (ACh). The authors confirmed that salt stress produced a reduction in chlorophyll biosynthetic intermediates, i.e., protoporphyrin-IX, Mg-photoporphyrin-IX, and protochlorophyllide, which were significantly enhanced in the presence of ACh. The stomatal closure is the first and imminent response to salinity stress, and it is usually related to a low leaf osmotic potential (Figure 1B) [34] as well as carbon assimilation and accumulation (Figure 2A,C). Lettuce treated with HES and (in particular) with HES + CA resulted in adjusting the carbon assimilation and the stomatal biochemical variation to control conditions. This is in line with previous studies, which revealed that applying phenolic compounds such as luteolin [35] or catechin [36] alleviates the negative effects of abiotic stress. Multivariate statistics and pathway analysis on metabolomics data confirmed that the application of exogenous phenolics shaped the lettuce metabolome although diversely in the presence or absence of stress. Interestingly, although all exogenous phenolics alleviated the negative effect of salt stress, the metabolomics analysis revealed a distinct mechanism of action once applied to the plants. Although CA and HES + CA showed the most distinct metabolomic profiles, HES-related modulation of metabolism seems to be effective in mitigating NaCl stress. 

Mei et al. [21] reported the protective role of CA against abiotic stress, in particular to the herbicide methyl viologen, in apple leaves. These authors suggested that CA reduced the degree of damage by improving the antioxidant capacity of leaves and thus the ability to deal with oxidative stress. It is known that CA is a direct scavenger of H_2_O_2_, even though these authors also observed an increased activity of antioxidant enzymes in leaves, including POD, CAT, and PPO (Mei et al., 2020). In this line, Wang et al. (2014) reported that exogenous CA applied to apple fruits provoked an increase in the activity of phenylalanine ammonia lyase (PAL) and cinnamate-4-hydrolase (C4H) and a decrease in polyphenol oxidase (PPO) and peroxidase (POD) [21]. The accumulation of phenolics following exogenous application of CA is also in agreement with our previous results [24]. Our current findings strengthen the ability of CA and, to a lesser extent, HES + CA to enhance the accumulation of phenylpropanoids.

Nevertheless, the strong and broad modulation of metabolism triggered by CA suggests an intricate network of responses that is not limited to phenolics. It is noteworthy that phytohormone regulation was involved, with NaCl-treated plants presenting a strong accumulation of 7′-hydroxyabscisate, the degradation product of abscisic acid (ABA), after the addition of CA and HES + CA. A similar accumulation was also observed for HES under non-stress conditions, suggesting that ABA was involved in the response exogenous phenolics irrespective from the salinity level. ABA is a phytohormone involved in abiotic stress signaling [37], improving stomatal resistance to control transpiration and CO_2_ uptake [38], and it can protect against oxidative damage by modulating the expression of antioxidants-related genes [39]. Moreover, it has been reported that ABA can enhance plant defense by increasing the secondary metabolism in plant systems [37].

Moreover, CA and HES + CA increased 2-hydroxy-5-oxoproline and 5-oxo-l-proline under salt-stress and pyrroline-5-carboxylate in the presence of the combined treatment (HES + CA). Proline is a key osmolyte that usually accumulates in response to adverse conditions, and it has several roles in stress adaptation, recovery, and signaling [40]. It is known that ABA modulates proline synthesis both on transcriptional and post-transcriptional levels [41]. The accumulation of proline under plant stress conditions is repressed by brassinosteroids [40]. Considering that brassinosteroids were mainly accumulated in salt-treated plants and, to a lesser extent in HES-treated plants, this may explain the fact that proline osmoprotection was a response characteristic for CA and HES + CA but not for salt and HES treatment.

In addition to ABA, several signal molecules have been found modulated after the addition of phenolics. Pheophorbide, an intermediate of chlorophyll degradation, has been recently proposed as a chloroplast metabolic status signaling molecule that is linked to jasmonic acid signaling [42]. Both pheophorbide and several JA-related compounds were modulated in our work, revealing a very intricate plant response. In this sense, differential responses in JAs were found between CA, HES + CA, and HES. HES strongly elicited methyl jasmonate in non-stressed plants and 3-oxo-2-(cis-2′-pentenyl)-cyclopentane-1-(E-octa-2-enoyl)-CoA in stressed-plants, which is a condition leading to the accumulation of secondary metabolites [43].

All three phenolic treatments seemed to positively modulate glucosinolate biosynthesis [44], especially the accumulation of the aliphatic glucosinolate glucomalcommin. Previous studies determined that glucosinolates are involved in the plant response to salt stress alleviating the symptoms and maintaining plant growth [45]. In particular, Martínez-Ballesta et al. [46] investigated the interaction between aliphatic glucosinolates and plant water balance under salt stress in Arabidopsis. Although little is known about the implication of glucosinolates in abiotic stress, especially in salt stress, these authors suggested that glucosinolates are involved in the plant response to salinity and connect this protective effect to the modulation of the aquaporins. It has been reported that sinigrin exogenously applied to *Brassica oleracea* increased movement through the symplastic water pathway and aquaporin content [46]. Moreover, the function of glucosinolates in plants seems to be more complex than expected. For instance, Chen et al. [47] showed that the suppression of aliphatic glucosinolates implies a disorder in proteins and metabolites involved in photosynthesis, oxidative stress, and hormone metabolism. This contributes to explaining why CA, HES, and the combined treatment (HES + CA) were able to maintain the water balance in plants, mitigating the negative effect of NaCl even though they provided distinct metabolic modulations. Noteworthy, glucosinolates are regulated by both ABA and JA [48]. The increase in brassinosteroids in NaCl-treated plants might explain the modulation of osmolytes such as betaine aldehyde and polyamines [49] in NaCl-treated plants, which are considered to provide physiological protection to proteins and other cell membranes against various oxidative stress [50].

## 4. Materials and Method

### 4.1. Plant Material and Experimental Design

**Figure 5:**
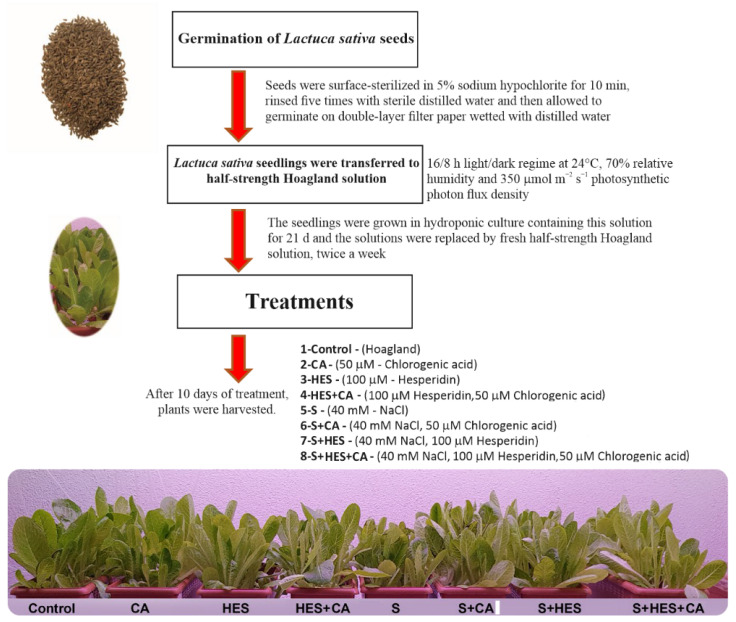
Workflow and experimental design of the germination, growth, and treatment of the seeds of *Lactuca sativa* L. Abbreviation: CA: Chlorogenic acid; HES: Hesperidin; S: Salt.

*Lactuca sativa* L. Yedikule M.5701 seeds were obtained from the Arzuman Seed Company (Karatay, Turkey). All plants were grown in hydroponic culture and optimum conditions management (16/8 h light/dark regime at 24 °C, 70% relative humidity and 350 μmol m^−2^ s^−1^ photosynthetic photon flux density) for 21 days (d). In the preliminary experiment, the levels of CA were treated as 25, 50, and 100 μM based on the previous studies reported by Mei et al. [21] and Liao et al. [51] during 10 d. Due to there being no study about exogenously treated HES to plants, 25, 50, 100, 150, and 200 μM HES were applied for 10 d. All treatments were applied to plants by adding to the half-strength Hoagland solution, and the solutions were replaced by fresh half-strength Hoagland solution, twice a week. After 10 days of treatments, it was determined that the appropriate CA and HES concentrations were 50 and 100 μM due to the absence of seedlings that can be analyzed in other concentrations. For the stress treatment, NaCl-induced salt stress was chosen depending on the study of Shin et al. [31] and Moncada et al. [52]. The experiment design and plant growth conditions are detailed and presented in Figure 5.

### 4.2. Determination of Growth, Water Content, and Osmotic Potential

The leaf growth (RGR) and relative water content (RWC) were measured according to the methods given by Hunt et al. [53] and Smart and Bingham [54], respectively. Leaf osmotic potential (Ψπ) was determined [55]. The experiments were repeated thrice independently, and each data point was the mean of six replicates.

### 4.3. Determination of Gas Exchange Parameters

Stomatal conductance (gs), carbon assimilation rate (A), transpiration rate (E), and intercellular CO_2_ concentration (Ci) were calculated with a portable gas exchange system (LCpro^+^; ADC, Hoddesdon, UK). The stomatal limitation value (Ls) was measured according to the formulae suggested by Ma et al. [56]. The experiments were repeated thrice independently, and each data point was the mean of five replicates.

### 4.4. Untargeted Metabolomics Analysis

Samples were extracted using 1.0 g of freeze-dried lettuce in 20 mL of 80% aqueous methanol with 0.1% formic acid (Merck KGaA, Darmstadt, Germany), using a Polytron^®^ PT 1200 E (Kinematica AG, Switzerland) homogenizer [24]. The extracts were centrifuged at 8000× *g* for 15 min, and untargeted metabolomics analysis was carried out using an Agilent ultra-high-pressure liquid chromatography coupled to a quadrupole-time-of-flight mass spectrometer (UHPLC-QTOF-MS; Agilent Technologies, Stevens Creek Blvd, Santa Clara, CA, USA), as previously reported [57]. In brief, chromatographic separation was carried out using an Agilent InfinityLab Poroshell 120 pentafluorophenyl (PFP) column (2.1 × 100 mm, 1.9 μm) (Santa Clara, CA, USA) and a binary mixture of water and acetonitrile acidified with 0.1% (*v/v*) formic acid as mobile phase (LC-MS grade, VWR, Milan, Italy). The mass spectrometer was operated in positive polarity and full-scan mode (100–1000 *m/z*) with a nominal resolution at 30,000 FWHM. The sequence injection was randomized, and Quality Control samples (QCs), made by pooling an aliquot of extract from each sample, were injected at the beginning of the sequence and every 6 sample injections. The Agilent software Profinder B.07 (Santa Clara, CA, USA) was used to align and annotate the raw metabolomic features according to the ‘find-by-formula’ algorithm, using the combination of monoisotopic accurate mass and the entire isotopic pattern, against the PlantCyc 12.6 database [58]. The approach adopted allowed a Level 2 of compound identification [59]. Compounds annotated in at least 75% of replicates within at least one treatment were retained for subsequent analysis.

### 4.5. Statistical Analysis

One-way analysis of the variance (ANOVA), followed by Tukey’s multiple comparisons (*p-*value *<* 0.05) of different physiological measurements, was carried out by using GraphPad Prism 9 (GraphPad Software, Inc., San Diego, CA, USA). Chemometric interpretation of metabolite profiles was performed using a Mass Profiler Professional B.12.06 from Agilent (Santa Clara, CA, USA), together with data transformation and normalization as reported in our previous work [60,61]. The unsupervised hierarchical cluster analysis (HCA) was carried out based on fold-change values. After that, supervised orthogonal projections to latent structures discriminant analysis (OPLS-DA) multivariate statistics analysis was carried out in SIMCA 16 (Umetrics, Malmo, Sweden). Therein, model fitness parameters (goodness of fit: R^2^Y; goodness of prediction: Q^2^Y; cross-validation: CV-ANOVA, *p* < 0.01) were determined. Permutation test (n = 200) and Hotelling’s T2 (95% and 99% confidence limit for the suspect and strong outliers, respectively) were also applied to validate and to investigate outliers, respectively. Then, the variable importance in projection (VIP > 1.3) approach was used to identify discriminant metabolites. 

Finally, the compounds that were statistically significant (*p-*value < 0.05 and FC ≥ 1.3) were uploaded into the Omic Viewer Pathway Tool of PlantCyc (Stanford, CA, USA) to identify the pathways and processes affected by treatments [62].

## 5. Conclusions

In a framework of sustainable agriculture, the ability of some natural compounds to mitigate abiotic stress is receiving large interest in the scientific community, with the aim of ensuring agricultural productivity under limiting conditions. With this regard, here, we investigated the ability of exogenous phenolics to mitigate salinity stress in lettuce. Our data indicate that different phenolics (chlorogenic acid, a phenolic acid, or hesperidin, a flavonoid) were able to mitigate physiological markers of plant stress, such as relative water content, osmotic potential, transpiration, and carbon assimilation rate. At the molecular level, chlorogenic acid imposed the broader metabolomic reprogramming, even though hesperidin showed interesting stress-mitigation peculiar traits. It is noteworthy that the response of lettuce to these different exogenous phenolics was broader than a mere oxidative stress mitigation, to involve phytohormones, osmolytes, and secondary metabolites such as glucosinolates. Moreover, our findings indicate that different phenolics provide distinct responses in lettuce, thus indicating peculiar effects likely related to the chemical diversity existing among the phenolic compounds.

## Figures and Tables

**Table 1:** Summary of different pathways activated in lettuce treated with 100 μM hesperidin, 50 μM chlorogenic acid, 100 μM HES + 50 μM CA. For each pathway highlighted under the different treatments, the number of compounds involved, average Log fold-change value (LogFC), and sum LogFC are provided. The LogFC values refer to a pairwise comparison between each treatment vs. control non-treated.

	Chlorogenic Acid			Hesperidin			Chlorogenic Acid + Hesperidin		
	N° of Compounds	Average LogFC	Sum LogFC	N° of Compounds	Average LogFC	Sum LogFC	N° of Compounds	Average LogFC	Sum LogFC
Amino acid	8	3.25	25.97	8	−4.68	−37.45	8	−1.21	−9.71
Nucleosides and Nucleotides	6	4.69	28.14	6	−0.54	−3.21	5	0.21	1.24
Fatty acid and Lipid	15	0.91	13.69	15	0.29	4.29	16	0.36	5.72
Amines and Polyamines	4	0.09	0.36	4	−4.13	−16.5	4	0.80	3.18
Carbohydrates	6	0.51	3.05	7	−3.54	−24.81	7	−1.66	−11.64
Secondary Metabolites	113	1.51	170.39	113	0.00	0.5	113	0.73	82.39
Cofactors, Electron Carriers	18	−0.23	−4.11	18	2.87	51.67	18	3.13	56.42
Hormones	5	0.01	0.04	5	−0.59	−2.95	5	−5.48	−27.38
Cell structures	8	0.28	2.21	8	2.06	16.47	9	2.13	19.15
Other biosynthesis	14	−0.85	−11.87	14	−1.93	−27.06	14	−0.21	−2.96

**Table 2:** Summary of different pathways activated on lettuce vegetables treated with 100 μM hesperidin, 50 μM chlorogenic acid, 100 μM HES + 50 μM CA and grown under 40 mM of sodium chloride (NaCl). For each pathway highlighted under different treatments, the number of compounds involved, average Log fold-change value (LogFC), and sum LogFC are provided. The LogFC values refer to a pairwise comparison between each treatment vs. control non-treated.

	Salt			Chlorogenic Acid			Hesperidin			Chlorogenic Acid + Hesperidin		
	N°of Compounds	Average FC	Sum FC	N° of Compounds	Average FC	Sum FC	N°of Compounds	Average FC	Sum FC	N°of Compounds	Average FC	Sum FC
Amino acid	8	0.60	4.76	8	2.83	22.64	8	1.88	15.04	8	7.73	61.87
Nucleosides and Nucleotides	10	5.90	58.95	9	0.10	0.90	9	−2.45	−22.01	10	3.74	37.37
Fatty acid and Lipid	20	0.15	3.00	20	−1.09	−21.82	20	1.23	24.50	20	−1.54	−30.81
Amines and Polyamines	2	9.10	18.20	2	1.42	2.84	2	−8.57	−17.13	2	10.57	21.13
Carbohydrates	8	2.18	17.45	8	−0.27	−2.18	8	−0.84	−6.68	8	4.41	35.24
Secondary Metabolites	186	0.81	151.33	185	0.59	109.57	177	0.41	72.95	185	2.88	533.05
Cofactors. Electron Carriers	14	3.36	47.02	14	3.21	44.89	14	−1.06	−14.89	14	8.29	116.00
Hormones	11	2.08	22.88	11	0.61	6.66	11	1.94	23.31	11	3.28	36.07
Cell structures	13	0.65	8.46	12	−2.58	−30.98	13	0.60	7.81	13	−5.09	−66.14
Other biosynthesis	17	1.54	26.20	17	4.17	70.94	16	0.49	7.78	17	5.15	87.54

## Data Availability

The raw data of this work are available as supplementary material. Further inquiries can be addressed to corresponding authors.

## Supplementary Materials

The following are available online, Figure S1: Morphological measurements, Figure S2: OPLS-DA validation models, Figure S3: Secondary metabolism pathways, Figure S4: Glucosinolates biosynthesis pathway, Table S1: Dataset, Table S2: Statistically significant metabolites of lettuces grown under 0 mM NaCl, Table S3: Statistically significant metabolites of lettuces grown under 40 mM NaCl, Table S4: VIP markers.

## Sample Availability

Not available.

## References

[1] Tandzi L.N., Mutengwa C.S. (2019). Estimation of Maize (*Zea mays* L.) yield per harvest area: Appropriate methods. Agronomy.

[2] Feizabadi A., Noormohammadi G., Fatehi F. (2020). Changes in growth, physiology, and fatty acid profile of rapeseed cultivars treated with vermicompost under drought stress. J. Soil Sci. Plant Nutr..

[3] Meena M., Divyanshu K., Kumar S., Swapnil P., Zehra A., Shukla V., Yadav M., Upadhyay R. (2019). Regulation of L-proline biosynthesis, signal transduction, transport, accumulation and its vital role in plants during variable environmental conditions. Heliyon.

[4] Sui N., Wang Y., Liu S., Yang Z., Wang F., Wan S. (2018). Transcriptomic and physiological evidence for the relationship between unsaturated fatty acid and salt stress in peanut. Front. Plant Sci..

[5] Dalal V.K., Tripathy B.C. (2012). Modulation of chlorophyll biosynthesis by water stress in rice seedlings during chloroplast biogenesis. Plant Cell Environ..

[6] Rossi S., Burgess P., Jespersen D., Huang B. (2017). Heat-induced leaf senescence associated with chlorophyll metabolism in bentgrass lines differing in heat tolerance. Crop Sci..

[7] Turan S., Tripathy B.C. (2015). Salt-stress induced modulation of chlorophyll biosynthesis during de-etiolation of rice seedlings. Physiol. Plant..

[8] Qin C., Ahanger M.A., Zhou J., Ahmed N., Wei C., Yuan S., Ashraf M., Zhang L. (2020). Beneficial role of acetylcholine in chlorophyll metabolism and photosynthetic gas exchange in Nicotiana benthamiana seedlings under salinity stress. Plant Biol..

[9] Bacellar I.O.L., Baptista M.S. (2019). Mechanisms of photosensitized lipid oxidation and membrane permeabilization. ACS Omega.

[10] Kaur P., Handa N., Kumar V., Bakshi P., Kalia R., Sareen S., Nagpal A., Vig A., Mir B., Bhardwaj R., Hasanuzzaman M., Fotopoulos V., Nahar K., Fujita M. (2019). Cross Talk Among Reactive Oxygen, Nitrogen and Sulfur During Abiotic Stress in Plants: Production, Metabolism, Signaling and Defebse Mechanism.

[11] Costa-Broseta Á., Perea-Resa C., Castillo M.-C., Ruíz M.F., Salinas J., León J. (2018). Nitric oxide controls constitutive freezing tolerance in Arabidopsis by attenuating the levels of osmoprotectants, stress-related hormones and anthocyanins. Sci. Rep..

[12] Kokotkiewicz A., Bucinski A., Luczkiewicz M. (2014). Light and temperature conditions affect bioflavonoid accumulation in callus cultures of *Cyclopia subternata* Vogel (honeybush). Plant Cell Tissue Organ Cult..

[13] Mahdavi V., Farimani M.M., Fathi F., Ghassempour A. (2015). A targeted metabolomics approach toward understanding metabolic variations in rice under pesticide stress. Anal. Biochem..

[14] Bistgani Z.E., Hashemi M., DaCosta M., Craker L., Maggi F., Morshedloo M.R. (2019). Effect of salinity stress on the physiological characteristics, phenolic compounds and antioxidant activity of *Thymus vulgaris* L. and Thymus daenensis Celak. Ind. Crops Prod..

[15] Parvin K., Nahar K., Hasanuzzaman M., Bhuyan M.B., Mohsin S.M., Fujita M. (2020). Exogenous vanillic acid enhances salt tolerance of tomato: Insight into plant antioxidant defense and glyoxalase systems. Plant Physiol. Biochem..

[16] Mekawy A.M.M., Abdelaziz M.N., Ueda A. (2018). Apigenin pretreatment enhances growth and salinity tolerance of rice seedlings. Plant Physiol. Biochem..

[17] Šamec D., Karalija E., Šola I., Vujčić Bok V., Salopek-Sondi B. (2021). The Role of polyphenols in abiotic stress response: The influence of molecular structure. Plants.

[18] Farah A., de Paula Lima J. (2019). Consumption of chlorogenic acids through coffee and health implications. Beverages.

[19] Tabeshpour J., Hosseinzadeh H., Hashemzaei M., Karimi G. (2020). A review of the hepatoprotective effects of hesperidin, a flavanon glycoside in citrus fruits, against natural and chemical toxicities. DARU J. Pharm. Sci..

[20] Kundu A., Vadassery J. (2019). Chlorogenic acid-mediated chemical defence of plants against insect herbivores. Plant Biol..

[21] Mei Y., Sun H., Du G., Wang X., Lyu D. (2020). Exogenous chlorogenic acid alleviates oxidative stress in apple leaves by enhancing antioxidant capacity. Sci. Hortic..

[22] Miler M., Živanović J., Ajdžanović V., Oreščanin-Dušić Z., Milenković D., Konić-Ristić A., Blagojević D., Milošević V., Šošić-Jurjević B. (2016). Citrus flavanones naringenin and hesperetin improve antioxidant status and membrane lipid compositions in the liver of old-aged Wistar rats. Exp. Gerontol..

[23] Adefegha S.A., Rosa Leal D.B., Olabiyi A.A., Oboh G., Castilhos L.G. (2017). Hesperidin attenuates inflammation and oxidative damage in pleural exudates and liver of rat model of pleurisy. Redox Rep..

[24] Zhang L., Martinelli E., Senizza B., Miras-Moreno B., Yildiztugay E., Arikan B., Elbasan F., Ak G., Balci M., Zengin G. (2021). The combination of mild salinity conditions and exogenously applied phenolics modulates functional traits in lettuce. Plants.

[25] Younis M.E., Hasaneen M.N., Ahmed A.R., El-Bialy D.M. (2009). Plant growth, metabolism and adaptation in relation to stress conditions. XXI. Reversal of harmful NaCl-effects in lettuce plants by foliar application with urea. Crop. Sci..

[26] Yildirim E., Ekinci M., Turan M., Dursun A., Kul R., Parlakova F. (2015). Roles of glycine betaine in mitigating deleterious effect of salt stress on lettuce (*Lactuca sativa* L.). Arch. Agron. Soil Sci..

[27] Kalhor M.S., Aliniaeifard S., Seif M., Asayesh E.J., Bernard F., Hassani B., Li T. (2018). Enhanced salt tolerance and photosynthetic performance: Implication of γ-amino butyric acid application in salt-exposed lettuce (*Lactuca sativa* L.) plants. Plant Physiol. Biochem..

[28] Bartha C., Fodorpataki L., Martínez-Ballesta M., Popescu O., Carvajal M. (2015). Sodium accumulation contributes to salt stress tolerance in lettuce cultivars. J. Appl. Bot. Food Qual..

[29] Simko I., Hayes R.J., Furbank R.T. (2016). Non-destructive phenotyping of lettuce plants in early stages of development with optical sensors. Front. Plant Sci..

[30] Sofo A., Lundegårdh B., Mårtensson A., Manfra M., Pepe G., Sommella E., De Nisco M., Tenore G.C., Campiglia P., Scopa A. (2016). Different agronomic and fertilization systems affect polyphenolic profile, antioxidant capacity and mineral composition of lettuce. Sci. Hortic..

[31] Shin Y.K., Bhandari S.R., Jo J.S., Song J.W., Cho M.C., Yang E.Y., Lee J.G. (2020). Response to salt stress in lettuce: Changes in chlorophyll fluorescence parameters, phytochemical contents, and antioxidant activities. Agronomy.

[32] Wang H., Tang X., Wang H., Shao H.-B. (2015). Proline accumulation and metabolism-related genes expression profiles in Kosteletzkya virginica seedlings under salt stress. Front. Plant Sci..

[33] Kalaji H.M., Govindjee, Bosa K., Kościelniak J., Żuk-Gołaszewska K. (2011). Effects of salt stress on photosystem II efficiency and CO_2_ assimilation of two Syrian barley landraces. Environ. Exp. Bot..

[34] Kosová K., Prášil I.T., Vítámvás P. (2013). Protein contribution to plant salinity response and tolerance acquisition. Int. J. Mol. Sci..

[35] El-Shafey N.M., AbdElgawad H. (2012). Luteolin, a bioactive flavone compound extracted from *Cichorium endivia* L. subsp. divaricatum alleviates the harmful effect of salinity on maize. Acta Physiol. Plant..

[36] Yiu J.-C., Tseng M.-J., Liu C.-W., Kuo C.-T. (2012). Modulation of NaCl stress in *Capsicum annuum* L. seedlings by catechin. Sci. Hortic..

[37] Bruňáková K., Petijová L., Zámečník J., Turečková V., Čellárová E. (2015). The role of ABA in the freezing injury avoidance in two Hypericum species differing in frost tolerance and potential to synthesize hypericins. Plant Cell Tissue Organ Cult..

[38] Zhang L., Gao M., Hu J., Zhang X., Wang K., Ashraf M. (2012). Modulation role of abscisic acid (ABA) on growth, water relations and glycinebetaine metabolism in two maize (*Zea mays* L.) cultivars under drought stress. Int. J. Mol. Sci..

[39] Guajardo E., Correa J.A., Contreras-Porcia L. (2016). Role of abscisic acid (ABA) in activating antioxidant tolerance responses to desiccation stress in intertidal seaweed species. Planta.

[40] Szabados L., Savouré A. (2010). Proline: A multifunctional amino acid. Trends Plant Sci..

[41] Pál M., Tajti J., Szalai G., Peeva V., Végh B., Janda T. (2018). Interaction of polyamines, abscisic acid and proline under osmotic stress in the leaves of wheat plants. Sci. Rep..

[42] Aubry S., Fankhauser N., Ovinnikov S., Pružinská A., Stirnemann M., Zienkiewicz K., Herrfurth C., Feussner I., Hörtensteiner S. (2019). Pheophorbide a may regulate jasmonate signaling during dark-induced senescence. Plant Physiol..

[43] Ho T.-T., Murthy H.N., Park S.-Y. (2020). Methyl jasmonate induced oxidative stress and accumulation of secondary metabolites in plant cell and organ cultures. Int. J. Mol. Sci..

[44] Lucini L., Rouphael Y., Cardarelli M., Canaguier R., Kumar P., Colla G. (2015). The effect of a plant-derived biostimulant on metabolic profiling and crop performance of lettuce grown under saline conditions. Sci. Hortic..

[45] Fatemi H., Carvajal M., Rios J.J. (2020). Foliar application of zn alleviates salt stress symptoms of pak choi plants by activating water relations and glucosinolate synthesis. Agronomy.

[46] Martinez-Ballesta M., Moreno-Fernández D., Castejon D., Ochando C., Morandini P., Carvajal M. (2015). The impact of the absence of aliphatic glucosinolates on water transport under salt stress in Arabidopsis thaliana. Front. Plant Sci..

[47] Chen Y.-Z., Pang Q.-Y., He Y., Zhu N., Branstrom I., Yan X.-F., Chen S. (2012). Proteomics and metabolomics of arabidopsis responses to perturbation of glucosinolate biosynthesis. Mol. Plant.

[48] Formisano L., Miras-Moreno B., Ciriello M., El-Nakhel C., Corrado G., Lucini L., Colla G., Rouphael Y. (2021). Trichoderma and phosphite elicited distinctive secondary metabolite signatures in zucchini squash plants. Agronomy.

[49] Sharma A., Shahzad B., Kumar V., Kohli S.K., Sidhu G.P.S., Bali A.S., Handa N., Kapoor D., Bhardwaj R., Zheng B. (2019). Phytohormones regulate accumulation of osmolytes under abiotic stress. Biomolecules.

[50] Giri J. (2011). Glycinebetaine and abiotic stress tolerance in plants. Plant Signal. Behav..

[51] Liao Y., Zeng L., Rao S., Gu D., Liu X., Wang Y., Zhu H., Hou X., Yang Z. (2020). Induced biosynthesis of chlorogenic acid in sweetpotato leaves confers the resistance against sweetpotato weevil attack. J. Adv. Res..

[52] Moncada A., Vetrano F., Esposito A., Miceli A. (2020). Fertigation management and growth-promoting treatments affect tomato transplant production and plant growth after transplant. Agronomy.

[53] Hunt R.W.G., Causton D.R., Shipley B., Askew A.P. (2002). A modern tool for classical plant growth analysis. Ann. Bot..

[54] Smart R.E., Bingham G.E. (1974). Rapid estimates of relative water content. Plant Physiol..

[55] Santa-Cruz A., Martinez-Rodriguez M.M., Perez-Alfocea F., Romero-Aranda R., Bolarin M.C. (2002). The rootstock effect on the tomato salinity response depends on the shoot genotype. Plant Sci..

[56] Ma L., Zhang H., Sun L., Jiao Y., Zhang G., Miao C., Hao F. (2012). NADPH oxidase AtrbohD and AtrbohF function in ROS-dependent regulation of Na^+^/K^+^ homeostasis in Arabidopsis under salt stress. J. Exp. Bot..

[57] Paul K., Sorrentino M., Lucini L., Rouphael Y., Cardarelli M., Bonini P., Reynaud H., Canaguier R., Trtílek M., Panzarová K. (2019). Understanding the Biostimulant Action of Vegetal-Derived Protein Hydrolysates by High-Throughput Plant Phenotyping and Metabolomics: A Case Study on Tomato. Front. Plant Sci..

[58] Schläpfer P., Zhang P., Wang C., Kim T., Banf M., Chae L., Dreher K., Chavali A.K., Nilo-Poyanco R., Bernard T. (2017). Genome-wide prediction of metabolic enzymes, pathways, and gene clusters in plants. Plant Physiol..

[59] Salek R.M., Steinbeck C., Viant M.R., Goodacre R., Dunn W.B. (2013). The role of reporting standards for metabolite annotation and identification in metabolomic studies. GigaScience.

[60] Ceccarelli A.V., Miras-Moreno B., Buffagni V., Senizza B., Pii Y., Cardarelli M., Rouphael Y., Colla G., Lucini L. (2021). Foliar application of different vegetal-derived protein hydrolysates distinctively modulates tomato root development and metabolism. Plants.

[61] Miras-Moreno B., Corrado G., Zhang L., Senizza B., Righetti L., Bruni R., El-Nakhel C., Sifola M.I., Pannico A., De Pascale S. (2020). The metabolic reprogramming induced by sub-optimal nutritional and light inputs in soilless cultivated green and red butterhead lettuce. Int. J. Mol. Sci..

[62] Caspi R., Dreher K., Karp P.D. (2013). The challenge of constructing, classifying, and representing metabolic pathways. FEMS Microbiol. Lett..

