# Supplementary material for: Metabolomics and Physiological Insights into the Ability of Exogenously Applied Chlorogenic Acid and Hesperidin to Modulate Salt Stress in Lettuce Distinctively
Source: Molecules. 2021 Oct 18;26(20):6291. doi: 10.3390/molecules26206291 (PMC8537907; doi:10.3390/molecules26206291)
Supplement: Supplementary file 1 [file molecules-26-06291-s001.zip › Supplementary Figure.pdf]

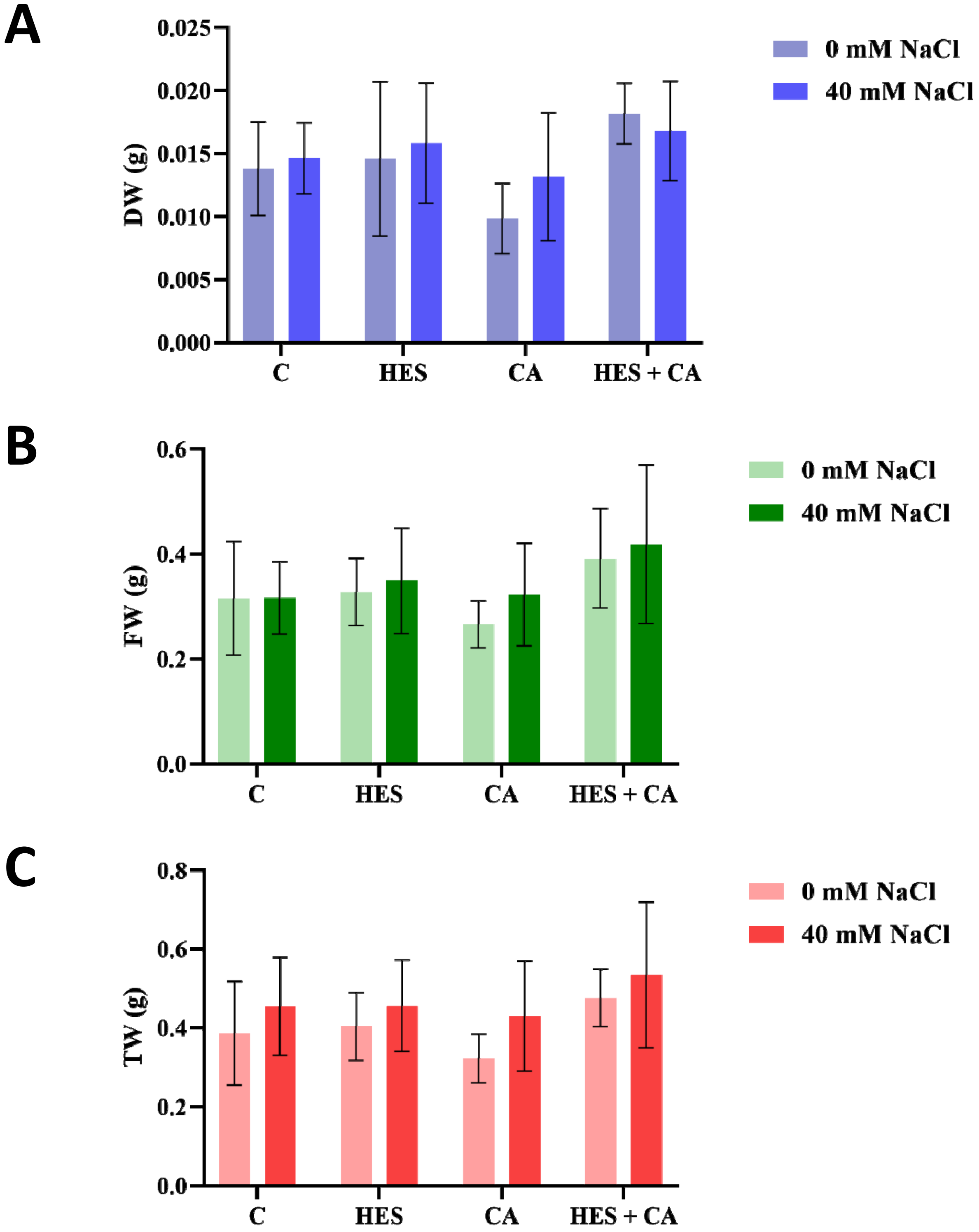

Figure S1. (**A**) Dry weight, (**B**) Fresh weight and (**C**) Turgid weight of *Lactuca sativa* after 10 days of treatments, the values are expressed as mean percentage ± standard deviation of four independent replicates. The lettuce plants were treated with: control no treated (C), 100 µM Hesperidin (HES), 50 µM chlorogenic acid (CA), 100 µM HES + 50 µM CA. Treatments were performed both under salt (40 mM NaCl) and non-salt (0 mM NaCl) stress. The statistical significance has been tested by a one-way ANOVA test with Tukey post-test multiple comparisons (p-value < 0.05) and significant differences between treatments are indicated by lowercase letters above each bar.

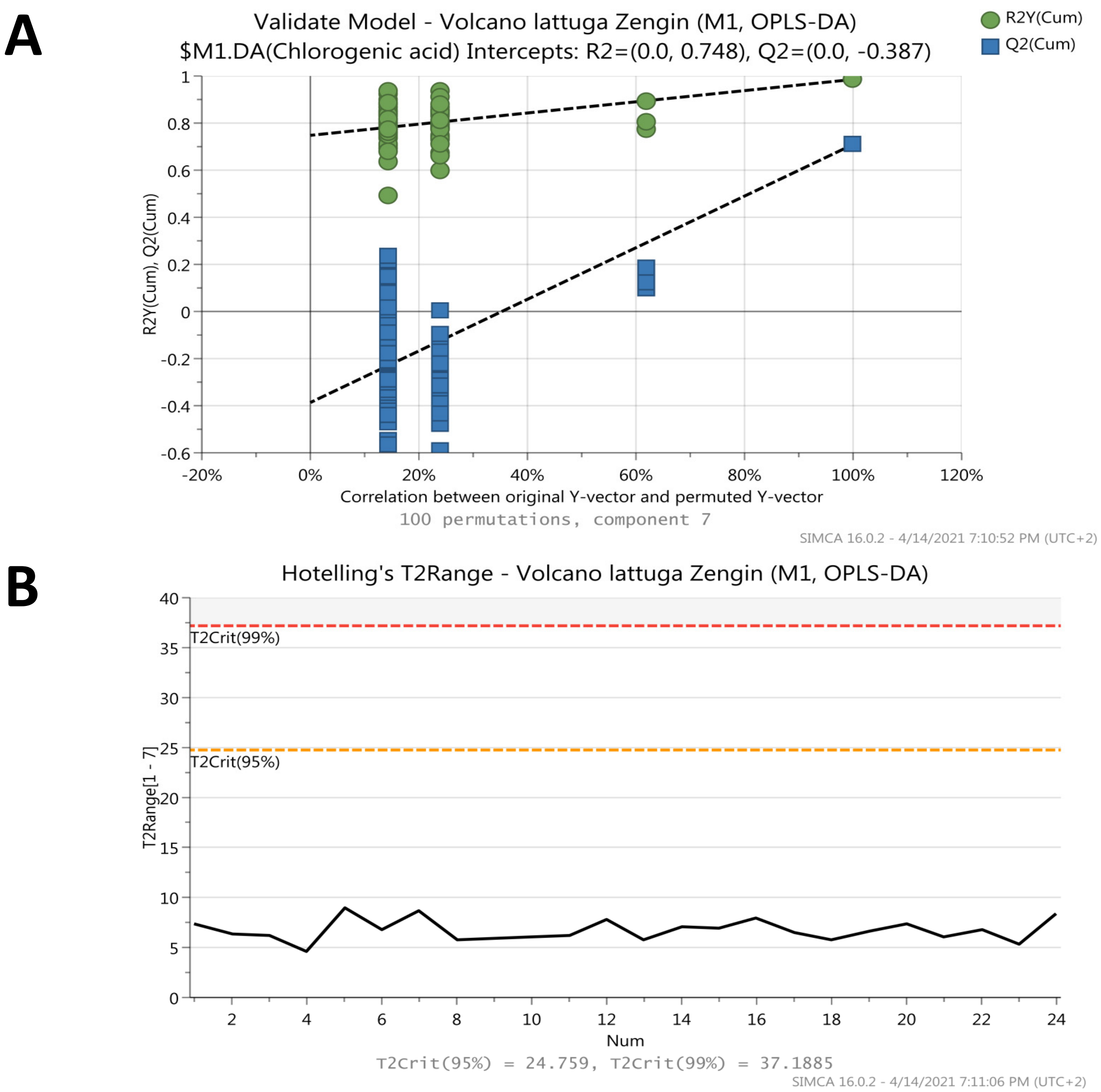

Figure S2. Validation of OPLS-DA discriminant model of *Lactuca sativa* after 10 days of treatments with 100 µM Hesperidin (HES), 50 µM chlorogenic acid (CA), 100 µM HES + 50 µM CA. Treatments were performed both under salt (40 mM NaCl) and non-salt (0 mM NaCl) stress. (**A**) permutation testing (N=100), (**B**) Hotelling's T2 using 95% and 99% confidence limits.

A

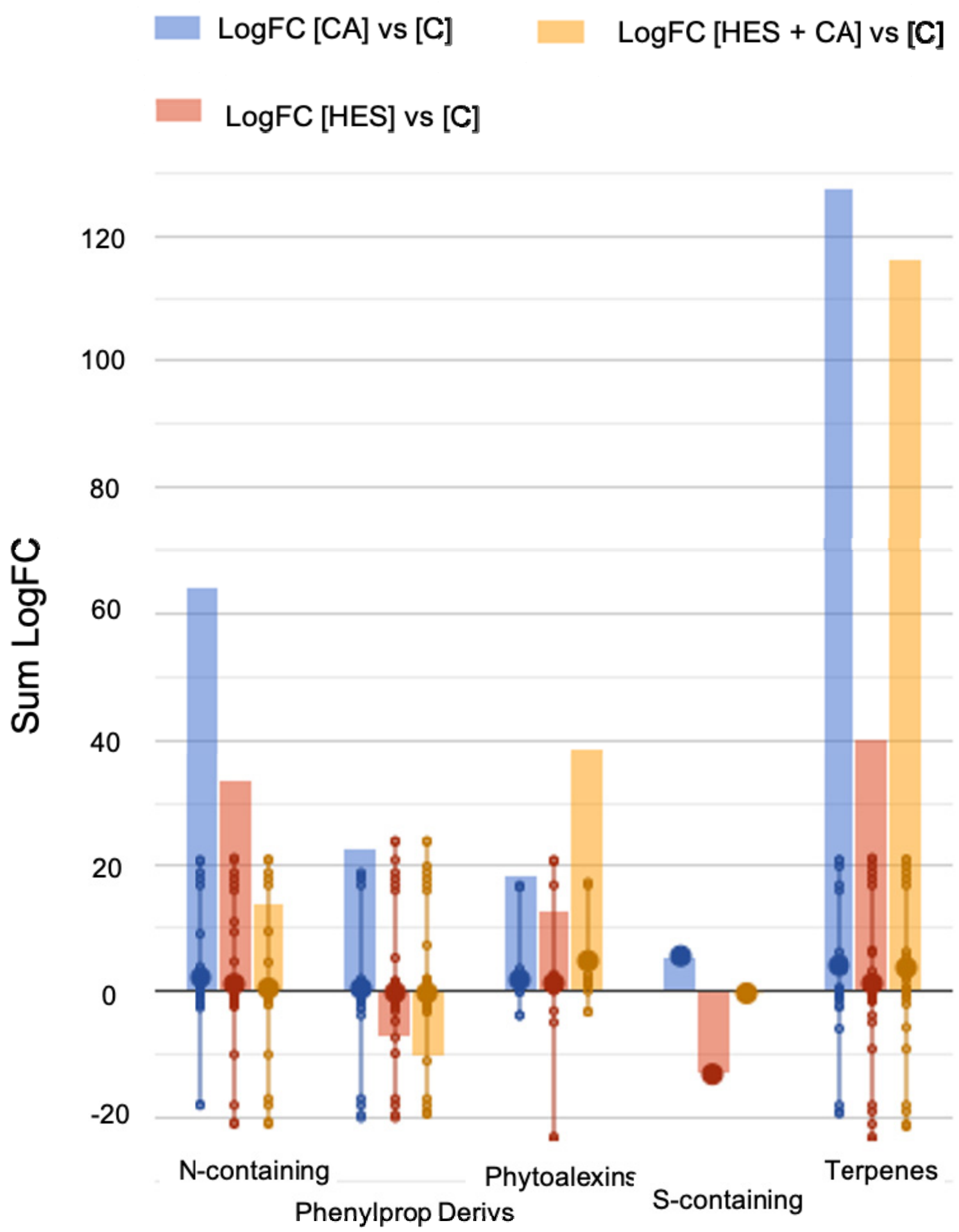

LogFC [CA] vs [C]
LogFC [HES + CA] vs [C]
LogFC [HES] vs [C]
Sum LogFC
120
100
80
60
40
20
0
-20
N-containing
Phenylprop Derivs
Phytoalexins
S-containing
Terpenes

B

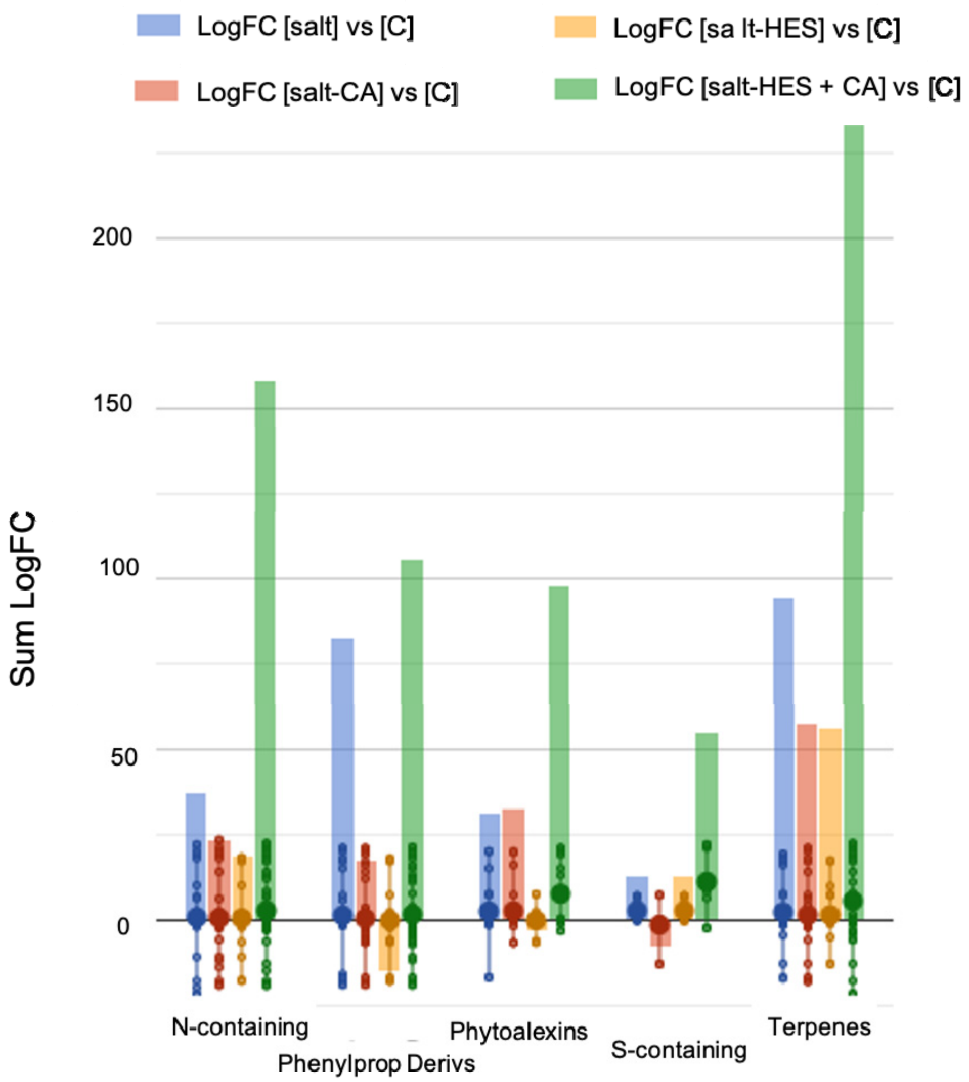

Figure S3. Secondary metabolism pathways carried out from lettuce vegetables after 10 days treatments with: 100 µM Hesperidin (HES), 50 µM chlorogenic acid (CA), 100 µM HES + 50 µM CA (Mix), grown under non-stressed condition (**A**) and salt-stressed condition (**B**). The dataset used to carry out the metabolomic analysis were produced through UHPLC-ESI/QTOF-MS and followed by a selection of compounds that passing Volcano Plot analysis (p-value < 0.05, fold-change > 1.3) and differential metabolites were loaded into PlantCyc Pathway Tool (https://www.plantcyc.org/). The abbreviated subcategory names on the x-axis correspond to: N-containing: nitrogen-containing; Phenylprop Derivs: phenylpropanoids and derivatives; S-containing: sulfur-containing.

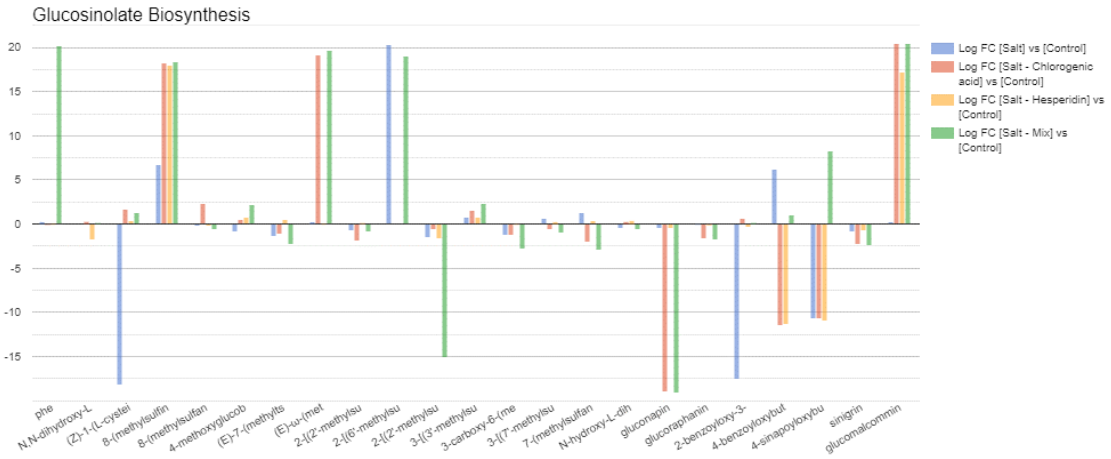

Figure S4. Glucosinolates biosynthesis pathway carried out from lettuce vegetables after 10 days treatments with: 100 µM Hesperidin (HES), 50 µM chlorogenic acid (CA), 100 µM HES + 50 µM CA (Mix), grown under salt-stressed condition. The dataset used to carry out the metabolomic analysis were produced through UHPLC-ESI/QTOF-MS and followed by a selection of compounds that passing Volcano Plot analysis (p-value < 0.05, fold-change > 1.3) and differential metabolites were loaded into PlantCyc Pathway Tool (https://www.plantcyc.org/).
