# Supplementary material for: Metabolomics and Physiological Insights into the Ability of Exogenously Applied Chlorogenic Acid and Hesperidin to Modulate Salt Stress in Lettuce Distinctively
Source: Molecules. 2021 Oct 18;26(20):6291. doi: 10.3390/molecules26206291 (PMC8537907; doi:10.3390/molecules26206291)
Supplement: Supplementary file 1 [file molecules-26-06291-s001.zip › Table S2.pdf]

Table S2. List of statistically significant metabolites that have passed the one-way analysis of variance (ANOVA) and with Log fold-change (LogFC) values greater than or equal to 1.3, obtained from lettuce samples grown under non-salinity condition. For each compound the category of the compound, the ontology of the compound, LogFC obtained by pairwise comparison between Chlorogenic Acid vs Control, Hesperidin vs Control, and Hesperidin + Chlorogenic Acid vs Control were reported.

Compound Name	Category	Ontology - parents of class	Log FC [Chlorogenic acid] vs [Control]	Log FC [Hesperidin] vs [Control]	Log FC [Hesperidin+Chlorogenic acid] vs [Control]
spermine	Amine and Polyamine Biosynthesis	Aliphatic-Alpha-Omega-Diamines	-0.06	-0.04	18.73
1,5-diazabicyclononane	Amine and Polyamine Degradation	Compounds	-0.06	17.96	0.05
1-(3-aminopropyl)-pyrrolinium	Amine and Polyamine Degradation	Compounds	-1.09	-0.84	-1.94
2-iminopropanoate	Amino Acid Biosynthesis	Imines // Enamines	5.61	-13.11	-0.29
L-alanine	Amino Acid Biosynthesis	Amino-Acids-20 // Non-polar-amino-acids // Neutral-Amino-Acids // L-Amino-Acids	-17.64	-17.57	-17.53
(2S,4S)-4-hydroxy-2,3,4,5-tetrahydrodipicolinate	Amino Acid Biosynthesis	Compounds	2.56	-7.84	-11.39
L-aspartyl-4-phosphate	Amino Acid Biosynthesis	Modified-Amino-Acids // L-Amino-Acids	-0.06	-0.04	17.46
5-(methylsulfanyl)-ribulose 1-phosphate	Amino Acid Biosynthesis	Compounds	1.77	0.49	0.9
1-(5-phospho-Œ≤-D-ribosyl)-AMP	Amino Acid Biosynthesis	Modified-Nucleosides	17.1	0.04	0.05
1-(5-phospho-Œ≤-D-ribosyl)-5-[(5-phosphoribosylamino)methylideneamino]imidazole-4-carboxamide	Amino Acid Biosynthesis	Carboxamides	17.23	0.04	0.05
1-(5-phospho-Œ≤-D-ribosyl)-ATP	Amino Acid Biosynthesis	Modified-Nucleosides	-0.6	0.54	1.04
2-oxoadipate	Amino Acid Degradation	2-Oxo-carboxylates	-16.76	-16.73	-16.65
UDP-Œ≤-L-arabinopyranose	Carbohydrate Biosynthesis	UDP-L-arabinopyranose	-0.62	-0.74	-0.51
(2E,14E,18E)-lycopatriene	Carbohydrate Biosynthesis	Hydrocarbons // Tetraterpenes	-18.19	-18.16	-18.05
dTDP-4-dehydro-6-deoxy-Œ±-D-glucopyranose	Carbohydrate Biosynthesis	NDP-4-dehydro-6-deoxy-D-glucose // DTDP-SUGARS // DTDP-4-DEHYDRO-6-DEOXY-D-GALACTOSE	-11.97	-6.2	-11.91

(2E,10E,14E,18E)-lycopatetraene	Carbohydrate Biosynthesis	Hydrocarbons // Tetraterpenes	-0.04	18.54	
formyl-CoA	Carbohydrates and Carboxylates Degradation	All-Coas	-18.61	-18.65	-18.57
1,18-octadecane-diol	Cell Structure Biosynthesis	Long-chain-alcohols // Diols	4.53	3.42	5.63
(2S)-ethylmalonyl-CoA	Cofactor, Carrier, and Vitamin Biosynthesis	All-Coas	16.96	17.48	17.32
4-aminobenzoate	Cofactor, Carrier, and Vitamin Biosynthesis	Benzoates	0.91	2.56	-0.08
7,8-diaminopelargonate	Cofactor, Carrier, and Vitamin Biosynthesis	Compounds	-0.86	-0.67	-0.88
1-deoxy-L-glycero-tetrulose 4-phosphate	Cofactor, Carrier, and Vitamin Biosynthesis	Compounds	-0.06	16.74	17.6
7,8-dihydropteroate	Cofactor, Carrier, and Vitamin Biosynthesis	Pteroates	-0.06	21.12	24.4
2-succinyl-5-enolpyruvoyl-6-hydroxy-3-cyclohexene-1-carboxylate	Cofactor, Carrier, and Vitamin Biosynthesis	Compounds	-0.17	-4.19	-4.02
homoglutathione	Cofactor, Carrier, and Vitamin Biosynthesis	TRIPEPTIDES // All-Glutathiones	-0.06	-0.04	19.46
(7,8-dihydropterin-6-yl)methyl diphosphate	Cofactor, Carrier, and Vitamin Biosynthesis	Pterins	-0.06	17.53	16.97
Œ±-tocotrienol	Cofactor, Carrier, and Vitamin Biosynthesis	Tocotrienols	-2.03	-2.32	-2.1
MoO2-molybdopterin cofactor	Cofactor, Carrier, and Vitamin Biosynthesis	Mo-molybdopterin-cofactor	-0.44	-0.65	-0.17
protoheme	Cofactor, Carrier, and Vitamin Biosynthesis	Heme-b	0.87	0.34	1.31
6-methoxy-3-methyl-2-all-trans-nonaprenyl-1,4-benzoquinol	Cofactor, Carrier, and Vitamin Biosynthesis	6Methoxy5Methyl2Polyprenyl14Benzoquinol	-0.84	-0.95	-0.7
6-(all-trans-decaprenyl)-2-methoxy-phenol	Cofactor, Carrier, and Vitamin Biosynthesis	2-Methoxy-6-polyprenyl-phenols	-0.06	-0.04	16.52
3-hydroxypropanoyl-CoA	Cofactor, Carrier, and Vitamin Biosynthesis	All-Coas	-0.19	-0.18	-17.19
3-methoxy-4-hydroxy-5-all-trans-decaprenylbenzoate	Cofactor, Carrier, and Vitamin Biosynthesis	3-Methoxy-4-Hydroxy-5-Polyprenylbenzoate	0.01	17.94	0.01
ubiquinol-10	Cofactor, Carrier, and Vitamin	Ubiquinols	0.73	1.74	2.6

	Biosynthesis				
UDP-2-N,3-O-bis[(3R)-3-hydroxytetradecanoyl]-Œ±-D-glucosamine	Fatty Acid and Lipid Biosynthesis	UDP-sugar	-0.06	16.56	0.05
sn-glycerol 3-phosphate	Fatty Acid and Lipid Biosynthesis	Glycerol-1-phosphate	-17.1	-17.08	-16.99
laurate	Fatty Acid and Lipid Biosynthesis	Medium-Chain-234-Saturated-Fatty-Acids // Saturated-Fatty-Acids	-0.69	-1.03	-1.18
3-dehydrosphinganine (C18)	Fatty Acid and Lipid Biosynthesis	CPD-22221	-1.68	-1.98	-2.45
digitoxigenin	Fatty Acid and Lipid Biosynthesis	3-Beta-Hydroxysterols // Cardenolides	-0.62	-0.74	-0.37
7-dehydrodesmosterol	Fatty Acid and Lipid Biosynthesis	Delta5-Delta7-Steroids // 3-Beta-Hydroxysterols	-0.96	-1	-0.57
4Œ±-methyl-5Œ±-cholesta-8,14,24-trien-3Œ≤-ol	Fatty Acid and Lipid Biosynthesis	Delta-14-steroids // 3-Beta-Hydroxysterols	-0.75	-0.82	-0.21
CDP-ethanolamine	Fatty Acid and Lipid Biosynthesis	Modified-Nucleotides // Pyrimidines	1.26	0.77	-0.77
4Œ±-carboxy-4Œ≤,14Œ±-dimethyl-9Œ≤,19-cyclo-5Œ±-ergost-24(241)-en-3Œ≤-ol	Fatty Acid and Lipid Biosynthesis	3beta-hydroxy-4alpha-carboxy-sterols	-0.73	-1.36	-0.16
CDP-choline	Fatty Acid and Lipid Biosynthesis	Nucleoside-Diphosphocholines	0.87	1.14	1.93
CDP-N-methylethanolamine	Fatty Acid and Lipid Biosynthesis	Compounds	17.14	18.26	17.96
4-(cytidine 5'-diphospho)-2-C-methyl-D-erythritol	Fatty Acid and Lipid Biosynthesis	CDP-SUGARS	-0.13	-9.13	-9.13
UDP-3-O-[(3R)-3-hydroxymyristoyl]-N-acetyl-Œ±-D-glucosamine	Fatty Acid and Lipid Biosynthesis	UDP-sugar	16.86		
malonyl-CoA	Fatty Acid and Lipid Biosynthesis	All-Coas	16.61	17.36	17.24
lauroyl-CoA	Fatty Acid and Lipid Biosynthesis	Medium-Chain-234-Saturated-acyl-CoAs	-16.66	-16.63	-16.54
11-hydroxydodecanoate	Fatty Acid and Lipid Biosynthesis	Medium-Chain-234-Saturated-Fatty-Acids // Omega-1-Hydroxy-Medium-Chain-Fatty-Acids	-19.38	-0.47	0.01
cis-12-sulfojasmonate	Hormone Biosynthesis	12-Hydroxyjasmonate-Sulfates	-0.9	-2.5	-8.35
isopentenyladenine-	Hormone	Adenine-Cytokinin-	-0.13	-0.74	-0.94

7-N-glucoside	Biosynthesis	Glucosides			
campest-4-en-3-one	Hormone Biosynthesis	3-Oxo-Delta-4-Steroids // Brassinosteroids	-0.83	-0.97	-0.61
N6-(Œî2-isopentenyl)-adenosine 5'-diphosphate	Hormone Biosynthesis	Compounds	19.54	18.83	0.05
16Œ±,17-epoxy gibberellin A4	Hormone Degradation	Gibberellins	0.39	0.06	0.82
selenate	Inorganic Nutrient Metabolism	Inorganic-Anions	-0.06	16.72	0.05
adenosine 5'-phosphoselenate	Inorganic Nutrient Metabolism	Compounds	-0.2	-0.57	-17.35
8-oxo-GMP	Nucleoside and Nucleotide Biosynthesis	All-Nucleosides	-0.05	-0.99	-0.99
N1-(5-phospho-Œ≤-D-ribosyl)glycinamide (GAR)	Nucleoside and Nucleotide Biosynthesis	Compounds	1.59	2.79	2.79
dTMP	Nucleoside and Nucleotide Biosynthesis	Pyrimidine-deoxyribonucleosides-5-P // Pyrimidines	-1.35	0.34	0.17
dCDP	Nucleoside and Nucleotide Biosynthesis	Pyrimidine-deoxyribonucleosides-5-PP // Pyrimidines	11.16	17.57	16.77
5'-phosphoribosyl-4-(N-succinocarboxamide)-5-aminoimidazole (SAICAR)	Nucleoside and Nucleotide Biosynthesis	Compounds	-0.48	-6.88	-1.54
dTTP	Nucleoside and Nucleotide Biosynthesis	Pyrimidine-deoxyribonucleosides-5-PPP // Pyrimidines	0.03	-17	-17
CTP	Nucleoside and Nucleotide Biosynthesis	Pyrimidine-ribonucleosides-5-PPP // Pyrimidines	17.19	-0.03	0.05
hypoxanthine	Nucleoside and Nucleotide Degradation	Purine-Bases // All-Nucleosides	-26.87	-26.84	-26.76
(S)-1-pyrroline-5-carboxylate	Other Biosynthesis	CPD-478	6.25	-0.04	17.21
dehydrospermidine	Other Biosynthesis	Aliphatic-Alpha-Omega-Diamines	-10.86	-0.81	-20.32
shikimate 3-phosphate	Other Biosynthesis	Compounds	-17.79	-0.19	-0.73
Mg-protoporphyrin	Other Biosynthesis	Porphyrins // Organometallics	1.29	1.29	2.07
3,8-divinyl chlorophyllide a	Other Biosynthesis	Chlorophyllides // Organometallics	-1.61	-20.1	-20.02
tetrahydrogeranylgeranyl chlorophyll a	Other Biosynthesis	Organometallics	0.3	-13.08	-5.25
4-hydroxybenzoyl-	Other Biosynthesis	All-Coas	-0.06	17.48	17.65

CoA					
chlorophyll b	Other Biosynthesis	Chlorophylls // Organometallics	5.41	1.59	5.43
cyclohexylamine	Other Pathways	Compounds	-0.09	-0.59	-0.76
sarcosine	Other Pathways	N-methylated-Amino-Acids	1.24	-17.57	1.78
2-furoate	Other Pathways	Compounds	-0.57	-19.83	-19.83
(2E)-2-hydroxypenta-2,4-dienoate	Other Pathways	Compounds	-16.69	-0.43	-8.45
L-2,4-diaminobutanoate	Other Pathways	L-Amino-Acids	-1.5	-19.29	-1.1
arsenite	Other Pathways	Compounds	13.78	4	0.79
methylmethanethiosulfonate	Other Pathways	Compounds	-6.97	-3.87	-7.46
4-guanidinobutyraldehyde	Other Pathways	Compounds	-0.05	-0.56	-2.72
triethanolamine	Other Pathways	Non-Metabolic-Compounds	-0.7	-0.89	-1.14
pteridine-ring	Other Pathways	Rings	-0.66	0.19	0.08
cinnamyl alcohol	Other Pathways	Compounds	0.28	0.2	0.74
3-chlorobenzaldehyde	Other Pathways	Aryl-Aldehyde // Chloroaromatic-Compounds	0.39	-20.6	1.19
phenylthiourea	Other Pathways	Compounds	-9.11	11.12	13.17
6-isobutyl-4-hydroxy-2-pyrone	Other Pathways	OXYGEN-HETEROCYCLES	-4.79	-0.81	-0.91
L-phosphinothricin	Other Pathways	Phosphinates	-0.95	-0.91	-8.08
(E)-geranylacetone	Other Pathways	Terpenoid-Derivatives	-0.37	2.99	-1.02
N-prenylagmatine	Other Pathways	Compounds	-0.62	-18.53	-0.28
(-)-cuparene	Other Pathways	Sesquiterpenes	-19.1	-0.27	-19.1
(-)-methyl jasmonate	Other Pathways	Methyl-Jasmonates	-0.06	19.5	0.05
4-phosphooxy-L-threonine	Other Pathways	Compounds	0.18	0.58	0.1
D,L-5-fluorotryptophan	Other Pathways	Compounds	-0.16	0.42	-0.14
sterone-ring	Other Pathways	Rings	-19.05	-19.05	-10.73
indolepropanol phosphate	Other Pathways	Indole-Derivatives	0.29	1.26	0.54
(S)-tetrahydroprotoberberine	Other Pathways	Compounds	-0.36	-0.58	-0.13
hypusine	Other Pathways	Amino-Acid-Derivatives // L-Amino-Acids	-8.37	1.74	10.69
N1-acetylspermine	Other Pathways	Aliphatic-N-Acetyl-Diamines	-0.1	-0.53	5.39
15-hydroxypentadecanoate	Other Pathways	Compounds	1.37	1.88	3.97

N-(4-aminobenzoyl)-L-glutamate	Other Pathways	Compounds	-1.34	-1.12	1.26
nitecapone	Other Pathways	C-nitro-compounds	-0.05	0.1	2.58
N-(4-hydroxybenzoyl)-L-glutamate	Other Pathways	Monocarboxylic-Acid-Amides	19.39	19.54	22.02
7-methylinosine	Other Pathways	Modified-Nucleosides	-0.44	-0.52	-0.57
1-monopalmitoylethylene glycol	Other Pathways	Compounds	-5.64	-1.8	-5.4
octyl Œ≤-D-glucopyranoside	Other Pathways	Glycolipids	-0.45	-1.16	-0.71
4-[(4E,6E)-3-oxo-7-phenylhepta-4,6-dien-1-yl]cyclohexa-3,5-diene-1,2-dione	Other Pathways	Compounds	0.77	-20.02	1.49
(4E,8E)-sphinga-4,8-dienine	Other Pathways	Sphingadienine	-1.46	-2.08	-2.03
di-homo-Œ≥-linolenate	Other Pathways	Omega-6-Fatty-Acids // Eicosatrienoates	1.03	-0.32	0.99
icosatetraenoate	Other Pathways	Long-Chain-234-Saturated-Fatty-Acids // Omega-3-Fatty-Acids	-0.76	-1.27	-1.63
sphinganine (C20)	Other Pathways	D-erythro-Shinganines	-1.36	-1.3	0.47
5'-(aminoiminomethyl)amino-5'-deoxyadenosine	Other Pathways	Compounds	-0.23	-0.95	-0.77
phytanate	Other Pathways	Saturated-Fatty-Acids // 3-Methyl-Saturated-Fatty-Acids	27.06	17.88	26.85
diphosphoglycolyl proline	Other Pathways	Compounds	0.01	-0.83	-0.46
2'-deoxyadenosine 3'-monophosphate	Other Pathways	Purine-deoxyribonucleotides // Deoxy-Ribonucleoside-3P	0.02	1.81	1.87
3'-ketolactose	Other Pathways	Carbohydrate-Derivatives	1.05	-17.89	-17.8
9-[6(RS)-C-carboxamido-5,6,7-trideoxy-Œ≤-D-ribo-octofuranosyl]-9H-purin-6-amine	Other Pathways	Compounds	-0.18	-1.03	0.56
guanosine 3'-monophosphate (3'-GMP)	Other Pathways	3-Phosphomonucleotides // All-Nucleosides	-0.06	-0.04	17.51
2-(3-benzoylphenyl)-3,5,7-	Other Pathways	Flavonol-Derivatives	-0.11	-1.47	-1.41

trihydroxychromen-4-one					
1,2-dioctanoyl-3-methyl-1,2,3-butanetriol	Other Pathways	Compounds	2.25	2.56	2.91
1-stearoyl-sn-glycerol	Other Pathways	1-Acyl-sn-glycerols	-0.81	-0.53	-0.23
7-O-Œ≤-D-glucosyl-7-hydroxyflavone	Other Pathways	Flavones	-0.33	2.76	2.83
5,5'-dithio-bis-2-nitrobenzoate	Other Pathways	C-nitro-compounds	16.85	16.23	16.63
3,5-dihydroxy-6,7-didehydro-12'-apo-Œ≤-caroten-12'-al	Other Pathways	12-apo-Carotenals	-0.01	0.02	-0.51
tetrahydrothiamine diphosphate	Other Pathways	ORGANOSULFUR	17.77	16.97	0.05
5'-(p-nitrophenyl)thioadenosine	Other Pathways	C-nitro-compounds	-6.69	-19.26	-19.26
2,4-diamino-6-ethyl-5,3'-(2-cyclohexylphenoxy)prop-1'-yloxypyrimidine	Other Pathways	Compounds	0.43	0.3	1.17
2'-methoxythiamine pyrophosphate	Other Pathways	Compounds	17.96	-0.04	0.05
scutellarin	Other Pathways	Flavones	0.77	-16.84	-16.75
8-hydroxy-dATP	Other Pathways	Modified-Nucleotides	17.23	-0.04	0.05
2-thiouridine 5'-triphosphate	Other Pathways	Compounds	-0.43	-0.62	-0.16
N10-(bromoacetyl)-5,8-dideazafolate	Other Pathways	Bromoaromtic-Compounds	-0.03	-16.89	-16.8
CDP-Œ±-D-glucose	Other Pathways	NDP-alpha-D-glucoses // CDP-SUGARS	-0.35	-0.51	-0.51
uridine-5'-diphosphate bromoacetol	Other Pathways	Bromoaromtic-Compounds // Pyrimidines	-0.5	-0.59	-0.17
all-trans-phytoene	Other Pathways	carotenoids	17.35	17.19	8.6
guanosine tetraphosphate	Other Pathways	Modified-Nucleotides	-0.6	-0.67	-0.49
pheophorbide b	Other Pathways	Chlorins	0.25	0.01	0.84
p-(bromoacetamido)phenyl uridyl pyrophosphate	Other Pathways	Bromoaromtic-Compounds	-0.79	-0.84	-0.7
cyclobis-(1,Üí6)-Œ±-nigerosyl	Other Pathways	Tetrasaccharides	-0.2	-1.47	-6.09
zaragozic acid C	Other Pathways	Compounds	16.88	18.03	17.8
all-trans-undecaprenyl group	Other Pathways	Polyprenol-Groups	5.62	17.1	-0.02
ubiquinone-9	Other Pathways	Ubiquinones	0.64	-0.05	-5.93
1,2-dipalmitoyl-phosphatidylglycero	Other Pathways	L-1-PHOSPHATIDYL-	10.91	18.14	18.41

l-phosphate		GLYCEROL-P			
phosphatidylglycerophosphate (dioctadec-9-enoyl(Z))	Other Pathways	L-1-PHOSPHATIDYL-GLYCEROL-P	18.43	18.75	18.02
cytochrome c	Other Pathways	Compounds	18.09	17.96	17.81
ferroheme o	Other Pathways	HEME_O	16.57	16.93	17.05
maltopentaose	Other Pathways	Linear-Malto-Oligosaccharides	-17.53	-0.17	0.18
ubiquinone-10	Other Pathways	Ubiquinones	-0.05	-0.05	-1.24
N-methylaminobutanal	Secondary Metabolite Biosynthesis	Compounds	9.1	9.45	9.51
propyl acetate	Secondary Metabolite Biosynthesis	Acetate-esters	-19.55	-19.53	-19.44
(2S)-methylbutanenitrile	Secondary Metabolite Biosynthesis	Nitriles	0.08	-0.45	0.39
hexanoate	Secondary Metabolite Biosynthesis	Medium-Chain-234-Saturated-Fatty-Acids // Saturated-Fatty-Acids	-0.66	-0.9	-1.16
tropinone	Secondary Metabolite Biosynthesis	Compounds	-2.56	-2.2	-2.01
2-phenylethylamine	Secondary Metabolite Biosynthesis	Aralkylamines	-2.68	-2.54	-2.08
Œ≥-coniceine	Secondary Metabolite Biosynthesis	ALKALOID	-17.79	-17.79	-17.79
4-coumaraldehyde	Secondary Metabolite Biosynthesis	Phenylpropanoids	-0.56	-3.12	-3.12
3,4-dihydrocoumarin	Secondary Metabolite Biosynthesis	Aromatics	-0.06	23.89	23.88
(+)-carvone	Secondary Metabolite Biosynthesis	Carvones	-0.31	-21.48	-21.39
(3E)-4,8-dimethylnona-1,3,7-triene	Secondary Metabolite Biosynthesis	Homoterpenoids	-19.33	-19.33	-19.33
(E)-7-(methyltsulfanyl)heptanal oxime	Secondary Metabolite Biosynthesis	CPD-19472	0.6	-0.82	0.53
3,6-dihydronicotine	Secondary Metabolite Biosynthesis	ALKALOID	-0.3	-10.01	-10.01
Œ≤-fenchene	Secondary Metabolite Biosynthesis	Compounds	6.21	6.46	6.35
2-tridecanone	Secondary Metabolite	Methylketones	-0.06	-0.04	17.14

	Biosynthesis				
ayapin	Secondary Metabolite Biosynthesis	Coumarins	18.02	-0.04	0.05
harmaline	Secondary Metabolite Biosynthesis	ALKALOID	0.1	0.65	0.78
trans-resveratrol	Secondary Metabolite Biosynthesis	Phytoalexins // Polyphenols	1.45	1.68	2.03
germacra-1(10),4,11(13)-trien-12-oate	Secondary Metabolite Biosynthesis	Sesquiterpenes	0.09	1.03	0.4
sinapaldehyde	Secondary Metabolite Biosynthesis	Phenylpropanoids	-0.34	0.65	0.72
lubimin	Secondary Metabolite Biosynthesis	Sesquiterpenes	-0.11	-4.88	
capsidiol	Secondary Metabolite Biosynthesis	Sesquiterpenes	-0.11	-23.49	
heliannuol C	Secondary Metabolite Biosynthesis	Sesquiterpenes	-0.5	-0.53	2.7
hemigossypol	Secondary Metabolite Biosynthesis	Sesquiterpenes	-0.2	0.02	2.25
2'-O-methylisoliquiritigenin	Secondary Metabolite Biosynthesis	Compounds	-0.02	0.77	1
abietatriene	Secondary Metabolite Biosynthesis	Diterpenes	-2.45	-1.29	-2.05
pseudobaptigenin	Secondary Metabolite Biosynthesis	Compounds	-3.76	-3.08	-3.24
(E)-1-(L-cystein-S-yl)-N-hydroxy-œâ-(methylsulfanyl)hexan-1-imine	Secondary Metabolite Biosynthesis	S-Substituted-L-Cysteines	3.87	4.67	4.62
phytol	Secondary Metabolite Biosynthesis	Long-Chain-Primary-Alcohols	4.48	3.23	5.53
1-(4-hydroxyphenyl)-3-(2,4,6-trihydroxyphenyl)propane-1,3-dione	Secondary Metabolite Biosynthesis	Open-tautomer-2-hydroxyflavanones	-0.5	-0.69	-1.6
shikonin	Secondary Metabolite Biosynthesis	Quinones // Secondary-Metabolites	2	1.53	1.85
ponciretin	Secondary Metabolite Biosynthesis	4-Methoxyflavanones	-2.57	-0.17	-2.6
16-epivellosimine	Secondary	Compounds	-1.2	-1.58	0.1

	Metabolite Biosynthesis				
7-hydroxy-4'5'-dimethoxyisoflavone	Secondary Metabolite Biosynthesis	Isoflavones	0.07	-0.63	-0.13
plaunotol	Secondary Metabolite Biosynthesis	Isoprenoid-Derivatives	-2.19	-3.84	-1.12
1,5,7-trihydroxy-6,8-dimethoxyanthraquinone	Secondary Metabolite Biosynthesis	Compounds	0.08	-0.69	0.22
(-)-phaseollin	Secondary Metabolite Biosynthesis	PTEROCARPANS	-0.06	20.75	0.05
hyperxanthone E	Secondary Metabolite Biosynthesis	Secondary-Metabolites // Aromatics	0.14	-1.11	-0.78
(L-cysteinylglycin-S-yl)(1H-indol-3-yl)acetonitrile	Secondary Metabolite Biosynthesis	CPD-6262 // Nitriles	3.6	2.72	2.68
sinapoyl-(S)-malate	Secondary Metabolite Biosynthesis	Compounds	-1.3	-9.79	-0.48
(S)-tetrahydrocolumbamine	Secondary Metabolite Biosynthesis	Compounds	-0.56	-20.76	-20.67
4-(methylsulfanyl)butyl-desulfoglucosinolate	Secondary Metabolite Biosynthesis	Desulfoaliphaticglucosinolates	0.24	-0.47	0.29
scopolin	Secondary Metabolite Biosynthesis	Compounds	0.29	-7.3	-0.6
2,3-dehydrokievitone	Secondary Metabolite Biosynthesis	Compounds	0.06	0.05	17.46
gluconapin	Secondary Metabolite Biosynthesis	alkenyl-glucosinolate	-0.09	-1.19	-1.11
3-hydroxypropyl-glucosinolate	Secondary Metabolite Biosynthesis	omega-hydroxyalkyl-glucosinolates	-0.2	-1.69	-1.22
sinigrin	Secondary Metabolite Biosynthesis	alkenyl-glucosinolate	0.18	-1.83	-0.9
3,7,3',5'-tetramethylmyricetin	Secondary Metabolite Biosynthesis	Flavonol-Derivatives	-0.45	-1.79	-1.54
dopaxanthin quinone	Secondary Metabolite Biosynthesis	ALKALOID	0.46	-0.69	-0.62
dopaxanthin	Secondary Metabolite Biosynthesis	ALKALOID	0.06	-1.33	-1.1
bixin dimethyl ester	Secondary Metabolite Biosynthesis	Ketocarotenoids // Carboxylic-esters	0.59	-0.82	-0.64

4-(methylsulfanyl)butyl-glucosinolate	Secondary Metabolite Biosynthesis	Omega-methylthio-alkyl-glucosinolates	0.07	-0.85	-1.16
2'-hydroxy 3,6,7,3',4'-pentamethylquercetagetin	Secondary Metabolite Biosynthesis	Flavonol-Derivatives	0.1	-1.18	-0.76
apigenin 7-O-Œ≤-D-glucoside	Secondary Metabolite Biosynthesis	Compounds	-0.06	19.17	19.67
2',3,4,4',6'-pentahydroxychalcone 4'-O-Œ≤-D-glucoside	Secondary Metabolite Biosynthesis	Chalcones	0.05	-0.78	-0.68
6-methylsulfinylhexyl-glucosinolate	Secondary Metabolite Biosynthesis	omega-methylsulfinylalkylglucosinolate	-2.09	-2.37	-2.1
glucomalcommin	Secondary Metabolite Biosynthesis	Aliphatic-glucosinolates	18.65	17.76	19.31
isorhamnetin 3, 4'-bisulfate	Secondary Metabolite Biosynthesis	Compounds	-0.06	16.34	16.48
patulitrin	Secondary Metabolite Biosynthesis	Flavonol-Derivatives	0.85	-16.68	-16.59
steviolmonoside	Secondary Metabolite Biosynthesis	Diterpenes	20.94	-0.04	0.05
eupatolitin 3-glucoside	Secondary Metabolite Biosynthesis	Flavonol-Derivatives	1.3	-0.23	0.33
2-benzoyloxy-3-butenylglucosinolate	Secondary Metabolite Biosynthesis	Compounds	-17.9	-17.87	-17.78
8-(methylsulfinyl)octyl-glucosinolate	Secondary Metabolite Biosynthesis	omega-methylsulfinylalkylglucosinolate	18.38	11.33	17.94
hypericin	Secondary Metabolite Biosynthesis	Dyes	17.09	-0.04	0.05
malonyldaidzin	Secondary Metabolite Biosynthesis	Compounds	0.8	0.98	1.41
emodin dianthrone	Secondary Metabolite Biosynthesis	Compounds	16.6	-0.04	0.05
2'-hydroxy 3,6,7,4'-tetramethylquercetagetin 3'-O-Œ≤-D-glucoside	Secondary Metabolite Biosynthesis	Flavonol-Derivatives	-1.12	-0.46	-0.28
pelargonidin 3-O-Œ≤-D-sambubioside	Secondary Metabolite Biosynthesis	Anthocyanins	1.1	-0.25	0.24
gomphrenin I	Secondary Metabolite Biosynthesis	ALKALOID	17.19	19.29	0.07

pelargonidin 3-O-(6''-O-malyl-Œ≤-D-glucoside)	Secondary Metabolite Biosynthesis	Anthocyanins	0.89	-0.56	-0.71
5'-demethoxy-6-methoxypodophyllotoxin 7-glucoside	Secondary Metabolite Biosynthesis	LIGNAN	-0.19	5.32	7.35
cyanidin 3-O-(Œ≤-D-xylosyl-(1,Üí2)-Œ≤-D-galactoside)	Secondary Metabolite Biosynthesis	Anthocyanins	-0.02	17.07	17.34
maysin	Secondary Metabolite Biosynthesis	Flavones	-0.87	-0.55	-1.26
kaempferol-3-O-Œ≤-D-glucopyranoside-7-O-Œ±-L-rhamnopyranoside	Secondary Metabolite Biosynthesis	Flavonol-3-O-Glucoside-7-O-Rhamnosides	-0.61	-0.62	-2.4
2-sinapoyloxy-3-butenylglucosinolate	Secondary Metabolite Biosynthesis	Compounds	-0.38	-0.43	-16.88
isovitexin 2''-O-rhamnoside	Secondary Metabolite Biosynthesis	Flavones	-0.14	-18.31	0.63
pelargonidin-3-O-rutinoside	Secondary Metabolite Biosynthesis	Anthocyanidin-3-O-beta-D-glucosides	-1.73	-1.66	-19.03
narirutin	Secondary Metabolite Biosynthesis	Compounds	-17.89	-17.77	-17.68
naringin	Secondary Metabolite Biosynthesis	Flavonone-rhamnosyl-glucosides	0.23	-4.73	-11.05
baccatin III	Secondary Metabolite Biosynthesis	Compounds	0.23	0.23	0.72
1,2-di-O-sinapoyl-Œ≤-D-glucose	Secondary Metabolite Biosynthesis	Compounds	-0.46	-18.26	-18.18
tricaffeoyl spermidine	Secondary Metabolite Biosynthesis	Amides	-1.86	-1.8	-1.24
3',5'-di-C-glucosylphloretin	Secondary Metabolite Biosynthesis	Compounds	0.73	0.33	1.16
quercetin 3, 3', 4', 7-tetrasulfate	Secondary Metabolite Biosynthesis	Flavonol-Derivatives	18.51	18.37	18.67
dalnigrein 7-O-Œ≤-D-apiofuranosyl-(1-6)-Œ≤-D-glucopyranoside	Secondary Metabolite Biosynthesis	Isoflavones	17.37		17.78
isorhamnetin 3-O-(6''-O-feruloyl)-glucoside	Secondary Metabolite Biosynthesis	Flavonol-Derivatives	-0.6	-0.77	-0.4
myricetin 3-O-gentiobioside	Secondary Metabolite Biosynthesis	Flavonol-3-O-D-Glucosides	-0.06	16.51	0.05

(+)-sesaminol 2-O-Œ≤-D-gentiobioside	Secondary Metabolite Biosynthesis	LIGNAN	-0.82	-17.64	-17.55
dihydroxyferuloyl-sinapoyl spermidine	Secondary Metabolite Biosynthesis	Compounds	20.5	17.29	17.43
N-debenzoyl-(3'-RS)-taxol	Secondary Metabolite Biosynthesis	ALKALOID	17.12	18.53	18.36
N-debenzoyl-(3'-RS)-2'-deoxytaxol	Secondary Metabolite Biosynthesis	ALKALOID	3.73	6.07	5.25
kaempferol 3-O-(3''-O-p-coumaroyl, 6''-O-feruloyl)-glucoside	Secondary Metabolite Biosynthesis	Flavonol-Derivatives	-0.06	16.82	0.05
stevioside	Secondary Metabolite Biosynthesis	Diterpenes	19.85	17.46	18.04
oleanolate 3-beta-D-glucuronoside-(3,1)-galactoside	Secondary Metabolite Biosynthesis	Triterpenes	16.46	19.09	19.62
monodeglucosyl des-acyl avenacin A	Secondary Metabolite Biosynthesis	Triterpenes	0.85	1.89	
Œ≤-D-gentiobiosyl Œ≤-D-glucosyl crocetin	Secondary Metabolite Biosynthesis	Beta-D-glucosides // Glycosyl-Esters	16.8	18.01	18.49
vincristine	Secondary Metabolite Biosynthesis	Organic-heteroptetracyclic-compounds	20.74	21.31	21.07
taxol	Secondary Metabolite Biosynthesis	Terpenoids	19.55	19.57	20.43
curcumin 4'-O-Œ≤-D-gentiotrioside	Secondary Metabolite Biosynthesis	POLYKETIDE // Phenols // Phenylpropanoids	-18.15	-18.17	-18.08
ternatin C5	Secondary Metabolite Biosynthesis	Anthocyanins	-16.75	-16.73	-16.64
(S)-3-hydroxy-3-methylglutaryl-CoA (HMG-CoA)	Secondary Metabolite Biosynthesis	All-Coas	-0.06		17.11
soyasaponin I	Secondary Metabolite Biosynthesis	Soyasaponins // Pentacyclic-triterpenoids	-5.87	-0.54	-5.67
(E,E)-piperoyl-CoA	Secondary Metabolite Biosynthesis	Compounds	-0.06	16.36	16.71
rebaudioside A	Secondary Metabolite Biosynthesis	Diterpenes	16.72	16.6	17.61
indolin-2-one	Secondary Metabolite Biosynthesis	Indole-Derivatives	2.65	1.82	1.17
dimethylsulfonio-2-hydroxybutanoate	Secondary Metabolite	Monocarboxylates // ORGANOSULFUR	-7.94	-0.45	-1.36

	Biosynthesis				
dioxindole-3-acetyl-3-O-beta-glucose	Secondary Metabolite Biosynthesis	Dioxindole-3-Acetyl-3-O-hexoses	0.45	0.43	-20.05
7-hydroxy-2-oxindole-3-acetate glucoside	Secondary Metabolite Biosynthesis	Compounds	0.45	0.43	-20.09
<i>N</i>-[β-glucosyl-(1,4)-β-glucosyl]-(oxindol-3-yl)acetyl-aspartate	Secondary Metabolite Biosynthesis	Indole-Derivatives // Modified-Amino-Acids	-19.45	-19.45	-19.45
benzylisothiocyanate	Secondary Metabolite Degradation	ISOTHIOCYANATES	-18.28	-0.89	-0.56
indole-3-carbinol	Secondary Metabolite Degradation	Indole-Phytoalexins	-0.98	-2.13	-2.13
2-benzyl-thiohydroximate-O-sulfate	Secondary Metabolite Degradation	THIOHYDROXIMATE-O-SULFATES	-0.06	16.75	17.34
7'-hydroxyabscisate	Secondary Metabolite Degradation	Abscisic-Acid-Derivative	-0.06	23.39	21.53
primeverose	Secondary Metabolite Degradation	Disaccharides	-7.74	15.74	-7.64
linalyl 6-O-Œ±-L-arabinopyranosyl-Œ≤-D-glucopyranoside	Secondary Metabolite Degradation	Monoterpenols // Carbohydrate-Derivatives	19.58	19.13	19.44
(glutathion-S-yl)-4-methoxy-3-indolylmethylisothiocyanate	Secondary Metabolite Degradation	Indole-Phytoalexins // S-Substituted-Glutathione	2.16	-0.84	1.37
D-myo-inositol (1,2,3,5,6) pentakisphosphate	Secondary Metabolite Degradation	D-myo-inositol-pentakisphosphates	-0.06		17.06
