# Supplementary material for: Metabolomics and Physiological Insights into the Ability of Exogenously Applied Chlorogenic Acid and Hesperidin to Modulate Salt Stress in Lettuce Distinctively
Source: Molecules. 2021 Oct 18;26(20):6291. doi: 10.3390/molecules26206291 (PMC8537907; doi:10.3390/molecules26206291)
Supplement: Supplementary file 1 [file molecules-26-06291-s001.zip › Table S3.pdf]

Table S3. List of statistically significant metabolites that have passed the one-way analysis of variance (ANOVA) and with Log fold-change (LogFC) values greater than or equal to 1.3, obtained from lettuce samples grown under salinity condition. For each compound the category of the compound, the ontology of the compound, LogFC obtained by pairwise comparison between salinity vs control, chlorogenic acid vs control, hesperidin vs control, and hesperidin + chlorogenic acid vs control were reported.

Compound Name	Category	Ontology - parents of class	Log FC [Salt] vs [Control]	Log FC [Salt - Chlorogenic acid] vs [Control]	Log FC [Salt - Hesperidin] vs [Control]	Log FC [Salt - Hesperidin + Chlorogenic Acid] vs [Control]
betaine aldehyde	Amine and Polyamine Degradation	Quaternary-Amines	0.35	0.09	-0.2	0.52
betaine aldehyde hydrate	Amine and Polyamine Degradation	Quaternary-Amines	-0.14	-0.09	-0.27	0.76
4-aminobutanal	Amine and Polyamine Degradation	Aliphatic-Omega-Amino-Aldehydes	19.38			20.42
1,5-diazabicyclononane	Amine and Polyamine Degradation	Compounds	18.65	18.52	0.03	-0.18
1-(3-aminopropyl)-4-aminobutanal	Amine and Polyamine Degradation	Aliphatic-Diamines	0.47	-0.77	-1.87	-0.96
1-(3-aminopropyl)-pyrrolinium	Amine and Polyamine Degradation	Compounds	0.57	-0.67	-1.78	-0.87
(S)-ureidoglycolate	Amine and Polyamine Degradation	Ureido-Compounds	0.78	-0.36	-0.32	-0.36
2-iminopropanoate	Amino Acid Biosynthesis	Imines // Enamines	6.92	-13.16	6.25	8.23
2-aminoprop-2-enoate	Amino Acid Biosynthesis	Enamines	0.26	-0.09	0.03	21.35
4-methyl-2-oxopentanoate	Amino Acid Biosynthesis	2-Oxo-carboxylates // Methyl-Branched-Fatty-Acids // Short-chain-fatty-acids	0.26	21.34	0.03	20.58
(2R)-2,3-dihydroxy-3-methylbutanoate	Amino Acid Biosynthesis	DIOH-ISOVALERATE	-10.79	-3.3	-7.34	-2.51
adenine	Amino Acid Biosynthesis	Purines // Purine-Bases	0.25	-0.91	-0.07	-1.29
D-erythro-imidazole-glycerol-phosphate	Amino Acid Biosynthesis	Compounds	-0.66	-0.78	-0.35	-2.86
2-hydroxy-5-(methylsulfanyl)-3-oxopent-1-enyl 1-phosphate	Amino Acid Biosynthesis	Compounds	0.06	19.63	-0.17	18.55
1-(5-phospho-Œ≤-D-ribosyl)-5-[(5-phosphoribosylamino)methylideneamino]imidazole-4-	Amino Acid Biosynthesis	Carboxamides	8.46	-0.09	16.66	-0.18

carboxamide						
(S)-3-hydroxy-isobutanoate	Amino Acid Degradation	3-HYDROXY-ISOBUTYRATE	0.26	-0.09	0.03	17.78
D-sedoheptulose 1,7-bisphosphate	Carbohydrate Biosynthesis	Sugar-Phosphate	0.26	16.28	0.03	17.33
Œ≤-L-fucose 1-phosphate	Carbohydrate Biosynthesis	Compounds	0.26	-0.09	0.03	17.97
6-kestotriose	Carbohydrate Biosynthesis	Levan	0.26	17.76	0.03	19.1
UDP-Œ≤-L-arabinopyranose	Carbohydrate Biosynthesis	UDP-L-arabinopyranose	-0.54	-0.43	-0.31	-0.94
dTDP-4-dehydro-6-deoxy-Œ±-D-glucopyranose	Carbohydrate Biosynthesis	NDP-4-dehydro-6-deoxy-D-glucose // DTDP-SUGARS // DTDP-4-DEHYDRO-6-DEOXY-D-GALACTOSE	-17.25	-17.6	-0.33	-17.69
(2E,14E,18E)-lycopatriene	Carbohydrate Biosynthesis	Hydrocarbons // Tetraterpenes	-1.57	-18.21	-0.43	-18.31
Œ≤-D-galactopyranose	Carbohydrates and Carboxylates Degradation	D-galactopyranose	0.16	-0.09	-0.01	21.55
formyl-CoA	Carbohydrates and Carboxylates Degradation	All-Coas	-18.27	-0.45	-0.42	-18.65
dodecane-1,7-diol	Cell Structure Biosynthesis	Diols	19.08		18.32	-0.19
1,18-octadecane-diol	Cell Structure Biosynthesis	Long-chain-alcohols // Diols	2.09	0.99	-1.21	1.17
18-hydroxyoleate	Cell Structure Biosynthesis	Omega-Hydroxy-long-Chain-Fatty-Acid	0.15	-1.04	-0.38	-1.92
Œ±,œâ-9Z-octadecenedioate	Cell Structure Biosynthesis	Alpha-omega-dicarboxylates	0.12	-1.76	-0.21	-3.21
2-(2,8-dihydroxytridecyl)-6-oxopyran-4-olate	Cell Structure Biosynthesis	POLY-HYDROXYLATED-TETRAKETIDE-PYRONE	1.11	-2.36	-7.14	-2.51
7-hydroxylauroyl-CoA	Cell Structure Biosynthesis	Medium-Chain-234-Saturated-acyl-CoAs // HYDROXY-ACYL-COA	0.35	-0.56	-0.16	-17.7
trichloroethanol	Chlorinated Compound Degradation	Compounds	-0.73	-17.83	-1.35	-17.93
trichloroethanol glucoside	Chlorinated Compound Degradation	Compounds	-0.71	-1.72	-0.12	-2.21
5-oxo-L-proline	Cofactor, Carrier, and Vitamin Biosynthesis	Compounds	0.26	17.42	0.03	17.27
4-aminobenzoate	Cofactor, Carrier, and Vitamin Biosynthesis	Benzoates	2.88	1.02	1.33	2.22
(R)-pantoate	Cofactor, Carrier,	Monocarboxylates	-13.89	-6.46	-0.8	7.91

	and Vitamin Biosynthesis					
7,8-diaminopelargonate	Cofactor, Carrier, and Vitamin Biosynthesis	Compounds	1.19	-17.51	-17.43	-17.86
1-deoxy-L-glycero-tetrulose 4-phosphate	Cofactor, Carrier, and Vitamin Biosynthesis	Compounds	0.26	17.96	0.03	17.87
4-amino-2-methyl-5-(diphosphooxymethyl)pyrimidine (HMP-PP)	Cofactor, Carrier, and Vitamin Biosynthesis	Compounds	0.26	-0.09	0.03	17.57
phylloquinol	Cofactor, Carrier, and Vitamin Biosynthesis	CPD-11501 // ETR-Quinols	-0.07	-1.7	0.04	-2.04
2-methoxy-6-(all-trans-nonaprenyl)phenol	Cofactor, Carrier, and Vitamin Biosynthesis	2-Methoxy-6-polyprenyl-phenols	0.99	-0.61	-0.48	1.34
all trans-decaprenyl-2-methoxy-6-1,4-benzoquinol	Cofactor, Carrier, and Vitamin Biosynthesis	2-Methoxy-6-Polyprenyl-14-Benzoquinols	1.35	1.15	1.89	0.47
3-methoxy-4-hydroxy-5-all-trans-decaprenylbenzoate	Cofactor, Carrier, and Vitamin Biosynthesis	3-Methoxy-4-Hydroxy-5-Polyprenylbenzoate	18.4	17.35	0.03	18.79
preuroporphyrinogen	Cofactor, Carrier, and Vitamin Biosynthesis	Compounds	-0.11	-0.01	0.35	-0.81
octanoyl-CoA	Cofactor, Carrier, and Vitamin Biosynthesis	Medium-Chain-234-Saturated-acyl-CoAs	-18.23	-18.23	-0.07	-18.64
epoxypheophorbide a	Cofactor, Prosthetic Group, Electron Carrier Degradation	Chlorins	-0.06	-1.02	0.08	-2.8
verdoheme	Cofactor, Prosthetic Group, Electron Carrier Degradation	Hemes	17.77	-0.09	0.03	-0.18
Œ±-hydroxyheme	Cofactor, Prosthetic Group, Electron Carrier Degradation	Hemes	-5.8	-0.22	0.1	-17.16
3R-hydroxy-lesqueroloyl-CoA	Fatty Acid and Lipid Biosynthesis	All-Coas	0.26	-0.09	16.56	-0.18
(3R,5Z,8Z,11Z,14Z,17Z)-3-hydroxydocosapentaenoyl-CoA	Fatty Acid and Lipid Biosynthesis	Compounds	-0.35	-0.36	-0.01	-1.17
O-phosphoethanolamine	Fatty Acid and Lipid Biosynthesis	Compounds	0.48	2.8	-17.15	3.73
laurate	Fatty Acid and Lipid Biosynthesis	Medium-Chain-234-Saturated-Fatty-Acids // Saturated-Fatty-Acids	0.69	-19.42	-0.62	-19.34
sphinganine (C18)	Fatty Acid and Lipid Biosynthesis	D-erythro-Shinganines	0.5	-0.69	0.03	-0.68
(9Z)-12,13-dihydroxyoctadeca-9-enoate	Fatty Acid and Lipid Biosynthesis	Long-Chain-234-Saturated-Fatty-Acids //	-0.25	-1.79	-0.36	-2.79

		Monounsaturated-fatty-acids				
allopregnanolone	Fatty Acid and Lipid Biosynthesis	3-Beta-Hydroxysterols // C21-Steroids	0.3	5.72	9.13	5.5
digitoxigenin	Fatty Acid and Lipid Biosynthesis	3-Beta-Hydroxysterols // Cardenolides	0.07	-0.89	-0.17	-1.8
7-dehydrodesmosterol	Fatty Acid and Lipid Biosynthesis	Delta5-Delta7-Steroids // 3-Beta-Hydroxysterols	-0.56	-2.4	-0.39	-4.21
squalene	Fatty Acid and Lipid Biosynthesis	Triterpenes	-1.74	-0.03	-1.4	-0.23
CDP-N-methylethanolamine	Fatty Acid and Lipid Biosynthesis	Compounds	17.53	16.89	17	11.97
CDP-ethanolamine	Fatty Acid and Lipid Biosynthesis	Modified-Nucleotides // Pyrimidines	-0.25	0.66	-0.34	0.5
4-(cytidine 5'-diphospho)-2-C-methyl-D-erythritol	Fatty Acid and Lipid Biosynthesis	CDP-SUGARS	-17.29	-17.29	-0.06	-5.85
1-16:0-2-18:2-diacylglycerol-trimethylhomoserine	Fatty Acid and Lipid Biosynthesis	CPD4LZ-25	3.7	-18.32	-1.01	-2.94
1-18:2-2-16:3-monogalactosyldiacylglycerol	Fatty Acid and Lipid Biosynthesis	Monogalactosyldiacylglycerols-34-5	-0.34	-0.71	0.29	-0.76
UDP-3-O-(3-hydroxymyristoyl)-Œ±-D-glucosamine	Fatty Acid and Lipid Biosynthesis	UDP-sugar	0.12	-1.22	0.14	-18.44
1-linoleoyl-2-linoleoyl-phosphatidylcholine	Fatty Acid and Lipid Biosynthesis	Phosphatidylcholines-36-4	3.82	-1.45	3.33	5.34
1-18:1-2-16:0-digalactosyldiacylglycerol	Fatty Acid and Lipid Biosynthesis	Digalactosyldiacylglycerols-34-1	-0.46	-0.57	-0.99	-1.6
lauroyl-CoA	Fatty Acid and Lipid Biosynthesis	Medium-Chain-234-Saturated-acyl-CoAs	-16.34	-0.18	0.32	-16.78
1-18:2-2-16:0-digalactosyldiacylglycerol	Fatty Acid and Lipid Biosynthesis	Digalactosyldiacylglycerols-34-2	-0.91	-0.84	-0.83	-1.42
trans-tuberonic acid glucoside	Hormone Biosynthesis	Jasmonate or derivatives	0.15	2.42	0.56	-1.59
3-oxo-2-(cis-2'-pentenyl)-cyclopentane-1-(E-buta-2-enoyl)-CoA	Hormone Biosynthesis	All-Coas	0.08	-0.64	-0.39	-18.37
N-acetyl-serotonin	Hormone Biosynthesis	Indole-Derivatives // N-Acetyl-2-Arylethylamines	0.26	-0.09	0.03	19.8
6-deoxocathasterone	Hormone Biosynthesis	Brassinosteroids	6.05	-7.48	6.83	-1.49
(22R,23R)-22,23-dihydroxy-campest-4-en-3-one	Hormone Biosynthesis	Brassinosteroids	1.12	-0.06	-0.35	0.06

(22R,23R)-22,23-dihydroxycampesterol	Hormone Biosynthesis	Brassinosteroids	-1.62	-0.78	-0.35	-0.93
3-dehydro-6-hydroxyteasterone	Hormone Biosynthesis	Brassinosteroids	1.15	-0.02	-0.34	0.08
N6-(Œî2-isopentenyl)-adenosine 5'-diphosphate	Hormone Biosynthesis	Compounds	17.57	17.92	0.03	16.84
3-oxo-2-(cis-2'-pentenyl)-cyclopentane-1-(3R-hydroxybutanoyl)-CoA	Hormone Biosynthesis	All-Coas	-0.63	-0.63	-0.18	-18.47
isopentenyl adenosine	Hormone Degradation	Nucleosides-Analogues	0.89	0.5	0.49	-0.12
16Œ±,17-epoxy gibberellin A4	Hormone Degradation	Gibberellins	1.15	-0.08	0.26	-0.09
26-hydroxybrassinolide	Hormone Degradation	Brassinosteroids	0.16	1.14	0.4	1.16
guanine	Nucleoside and Nucleotide Biosynthesis	Purines // Purine-Bases	0.06	-1.08	-0.27	-1.57
orotate	Nucleoside and Nucleotide Biosynthesis	Monocarboxylates // Pyrimidines	19.16			18.63
N1-(5-phospho-Œ≤-D-ribosyl)glycinamide (GAR)	Nucleoside and Nucleotide Biosynthesis	Compounds	-0.62	-0.55	1.53	-0.12
2-(formamido)-N1-(5-phospho-Œ≤-D-ribosyl)acetamidine (FGAM)	Nucleoside and Nucleotide Biosynthesis	Compounds	2.16	3.18	0.22	3.18
adenosine 5'-phosphoramidate	Nucleoside and Nucleotide Biosynthesis	CPD0-2580	1.84	2.32	0.61	1.98
GDP	Nucleoside and Nucleotide Biosynthesis	Purine-ribonucleosides-5-PP // All-Nucleosides	17.52	16.42	0.04	17.04
5'-phosphoribosyl-4-(N-succinocarboxamide)-5-aminoimidazole (SAICAR)	Nucleoside and Nucleotide Biosynthesis	Compounds	-0.19	-18.45	-18.36	-18.55
dTTP	Nucleoside and Nucleotide Biosynthesis	Pyrimidine-deoxyribonucleosides-5-PPP // Pyrimidines	1.05	-0.07	-5.73	0.67
CTP	Nucleoside and Nucleotide Biosynthesis	Pyrimidine-ribonucleosides-5-PPP // Pyrimidines	17.72	0.04	0.02	17.4
cyclic ADP-ribose	Nucleoside and Nucleotide Biosynthesis	ADP-RIBOSE-P-OR-NOP	-16.81	-0.42	-0.24	-0.79

(S)-1-pyrroline-5-carboxylate	Other Biosynthesis	CPD-478	19.2	19.33	19.52	20.79
(S)-1-pyrroline-5-carboxylate	Other Biosynthesis	Unclassified-Compounds				17.71
2-naphthol	Other Biosynthesis	Compounds	0.26	22.36	0.03	21.49
1-naphthol	Other Biosynthesis	Compounds	1.24	0.71	-0.27	-0.16
dehydrospermidine	Other Biosynthesis	Aliphatic-Alpha-Omega-Diamines	0.38	-0.74	-20.29	-0.94
2-[(2'-methylsulfanyl)ethyl]maleate	Other Biosynthesis	CPD-19484	-0.29	-1.38	-1.08	-0.81
3-[(2'-methylsulfanyl)ethyl]malate	Other Biosynthesis	CPD-19483	0.26	18.54	0.03	18.13
2-[(2'-methylsulfanyl)ethyl]malate	Other Biosynthesis	CPD-19482	8.82	18.32		17.91
chlorophyllide a	Other Biosynthesis	Chlorophyllides // Organometallics	0.57	1.31	1.12	1.41
8'-(3,4-dihydroxy-5-alkenyl)phenyl-3-[9'E,11'E,13'Z-pentadecatrienyl]catechol	Other Biosynthesis	Catechols	-0.07	-0.7	-1.43	0.92
71-dihydroxychlorophyllide a	Other Biosynthesis	Chlorophyllides // Organometallics	-19.12	-1.22	0.57	-5.55
dihydrogeranylgeranyl chlorophyll a	Other Biosynthesis	Organometallics	0.11	1.42	-0.28	1.56
tetrahydrogeranylgeranyl chlorophyll a	Other Biosynthesis	Organometallics	1.28	-0.62	0.98	-13.16
chlorophyll a	Other Biosynthesis	CPD-18899	1.05	2	0.17	1.09
chlorophyll b	Other Biosynthesis	Chlorophylls // Organometallics	5.44	4.87	2.08	-3.91
3-cyano-L-alanine	Other Degradation	Nitriles	-0.65	-1.19	0.06	-0.09
(E)-2-pentenal	Other Pathways	Unsaturated-aldehydes	1.67	1.67	2.05	1.26
thiosulfate	Other Pathways	Inorganic-Anions	9.15	-9.35	-0.41	9.87
cyclohexylamine	Other Pathways	Compounds	0.51	0.11	-0.46	-0.18
2-pyrrolidone	Other Pathways	Compounds	-19.74	-0.62	-0.11	0.19
butanoate	Other Pathways	Saturated-Fatty-Acids // Short-Chain-234-Saturated-Fatty-Acids	19.58			
N-isopropylformamide	Other Pathways	Compounds	0.26	20.37	0.03	19.55
2-furoate	Other Pathways	Compounds	-0.16	-0.26	-0.15	-19.93
dioxane	Other Pathways	Compounds	0.1	-18.35	-0.18	-18.35
enol-oxaloacetate	Other Pathways	C4-dicarboxylates	0.72	-0.57	0.39	-0.57
(R,R)-2,3-butanediol	Other Pathways	2-3-Butanediols	0.21	-0.1	0.02	19.12
2-(2-methylpyridin-3-yl)ethanol	Other Pathways	Pyridines	0.75	-1.81	-0.43	-2.97

L-2,4-diaminobutanoate	Other Pathways	L-Amino-Acids	0.99	-1.53	-1.07	-2.66
threose	Other Pathways	Aldotetraoses	-0.02	-2.95	0.12	-3.41
D-allothreonine	Other Pathways	ALLO-THR	22.22	-0.07	0.06	21.51
pyruvic oxime	Other Pathways	Compounds	0.19	18.35		5.45
arsenite	Other Pathways	Compounds	13.82	3.06	-6.31	1.9
methylmethanethiosulfonate	Other Pathways	Compounds	-17.07	-12.12	-10.7	-5.44
heptanoate	Other Pathways	Medium-Chain-234-Saturated-Fatty-Acids // Saturated-Fatty-Acids	-0.08	3.96	-0.05	4.33
triethanolamine	Other Pathways	Non-Metabolic-Compounds	0.41	-1.92	-0.79	-3.37
pteridine-ring	Other Pathways	Rings	0.14	1.91	-0.05	2.87
chromate	Other Pathways	Inorganic-Anions	-0.04	-0.58	-0.94	0.14
3-phenylpropanoate	Other Pathways	Aromatic-Acid // Monocarboxylates // Phenylalkanoates // Phenylpropanoids	3.76	1.26	2.62	-0.24
(1R)-trans-chrysanthemate	Other Pathways	Compounds	0.26	-0.09	0.03	19.29
aldehydo-L-arabinose	Other Pathways	L-ARABINOSE	0.22	1.96	-0.36	2.92
x-pyrophosphate-group	Other Pathways	Simple-Groups	0.08	-0.25	-0.1	-0.59
2-deoxy-D-galactose	Other Pathways	Modified-Sugars	0.26	21.3	0.03	21.08
thioguanine	Other Pathways	Thiopurines	0.26	17.22	0.03	-0.18
isophthalate	Other Pathways	dicarboxylate	0.26	19.21	0.03	19.86
2-hydroxy-5-oxoproline	Other Pathways	Modified-Amino-Acids	-0.1	2.85	-0.12	6.28
iodate	Other Pathways	Compounds	-0.4	-18.84	-18.84	-18.84
cumene hydroperoxide	Other Pathways	Alkyl-Hydro-Peroxides	-0.12	-0.46	-0.74	-0.59
decan-2-ol	Other Pathways	Secondary-Alcohols // Medium-Chain-Alcohols	0.26	0.01	16.39	0.01
fosmidomycin	Other Pathways	Antibiotics // Organophosphonates	7.09	6.14	4.36	8.23
difluoromethylornithine	Other Pathways	Compounds	0.63	-3.49	-0.48	-3.49
Hg2+	Other Pathways	Post-Transition-Metal-Cations // Divalent-Inorganic-Cations	0.01	-1.27	0.23	-0.41
D,L- Œ≥-hydroxyphosphinothricin	Other Pathways	Compounds	-1.09	-1.18	-0.15	-2.06
N-prenylagmatine	Other Pathways	Compounds	-18.53	-18.53	-18.53	-18.53
tetradecan-1-ol	Other Pathways	Long-Chain-Primary-Alcohols // Long-chain-alcohols	0.47	-0.93	-0.45	-1.24

(-)-cuparene	Other Pathways	Sesquiterpenes	-10.3	-19.25	-19.17	-19.17
(R)-lipoate	Other Pathways	Cofactors	0.32	13.52	-0.55	-6.58
(3R)-3-hydroxy-2-oxo-4 phosphooxybutanoate	Other Pathways	2-Oxo-carboxylates	0.01	-0.45	-0.26	-0.84
(2S)-2-amino-3-oxo-4-phosphooxybutanoate	Other Pathways	Compounds	0.26	19.7	17.37	20.7
4-phosphooxy-L-threonine	Other Pathways	Compounds	-0.1	0.14	0.62	0.09
cycloheptadienyl	Other Pathways	Compounds	0.09	1.77	1.07	3.55
D,L-5-fluorotryptophan	Other Pathways	Compounds	-20.72	-0.4	-0.38	-0.1
L-leucyl-L-leucine	Other Pathways	Dipeptides-Of-L-Amino-Acids	0.97	-1.77	0.14	-1.45
myristate	Other Pathways	Long-Chain-234-Saturated-Fatty-Acids // Saturated-Fatty-Acids	-0.31	0.59	0.22	-0.98
sterone-ring	Other Pathways	Rings	-0.2	-19.04	-1.7	-19.04
2-deoxy-6-phospho-D-gluconate	Other Pathways	Compounds	-1.21	1.3	0.26	0.02
L-cystine	Other Pathways	cystine // Non-standard-amino-acids	-0.06	1.78	-1.1	3.05
7-hydroxyflavone	Other Pathways	Flavones	-1.41	-1.1	0.06	-3
o-topolin	Other Pathways	Compounds	0.26	20.85	0.03	20.56
Œ±-oxo-1-carboxy-4-tetrahydrothiopyran propanoate S-oxide	Other Pathways	Compounds	-0.25	-0.69	-0.57	-1.63
2-amino-3-(phosphomethylphosphinyl)propanoate	Other Pathways	Compounds	0.28	2.32	0.43	3.73
N-(4-hydroxybenzoyl)-L-glutamate	Other Pathways	Monocarboxylic-Acid-Amides	0.26	19.01	0.03	19.02
indolepropanol phosphate	Other Pathways	Indole-Derivatives	0.67	1.08	0.79	1.11
Œ±-D-glucosamine 1-phosphate	Other Pathways	Sugar-Phosphate	-8.45	10.66	8.69	11.56
5-methylcytidine	Other Pathways	Compounds	-21.75	-1.77	-3.54	-3.06
15-hydroxypentadecanoate	Other Pathways	Compounds	2.17	0.03	-0.51	-17.34
pentachlorophenol	Other Pathways	Chloroaromatic-Compounds	18.13	17.97	17.38	18.36
risedronate	Other Pathways	Compounds	0.26	-0.09	0.03	16.46
7-methylinosine	Other Pathways	Modified-Nucleosides	0.04	-0.7	-0.3	-1.53
stearidonate	Other Pathways	Long-Chain-234-Saturated-Fatty-Acids // Omega-3-	0.31	-1.37		-2.7

		Fatty-Acids				
2',4,4',6'-tetrahydroxychalcone	Other Pathways	Chalcones	-0.97	-0.89	0.26	-1.64
(9Z,12E)-octadecadienoate	Other Pathways	Long-Chain-234-Saturated-Fatty-Acids // Polyunsaturated-fatty-acids	0.17	-0.71	-0.06	-1.31
(1,4)-Œ≤-xylobiose	Other Pathways	1-4-D-xylooligosaccharides // Disaccharides	-21.29	-12.2	-21.45	-1.86
8-oxo-deoxyguanosine	Other Pathways	All-Nucleosides	0.26	-0.09	0.03	21.58
L-threo-sphinganine (C18)	Other Pathways	DIHYDRO-SPHINGOSINE	-21.41	-0.69	0.03	-0.68
cetyltrimethylammonium	Other Pathways	Organic-Cations // Quaternary-Amines	0.01	-0.61	-0.03	-0.98
scutellarein	Other Pathways	Flavones	-0.66	-20.94	-20.82	-2.15
octyl Œ≤-D-glucopyranoside	Other Pathways	Glycolipids	0.17	-1.59	-0.35	-1.66
phytanate	Other Pathways	Saturated-Fatty-Acids // 3-Methyl-Saturated-Fatty-Acids	23.27	16.66	-0.05	20.69
dihydroisopteroate	Other Pathways	Pteroates	0.26	-0.09	0.03	20.32
9,10-epoxy-12-cis-octadecenoate	Other Pathways	Epoxy-fatty-acids	-0.14	-1.58	-0.31	-2.66
herbicide 14A	Other Pathways	Herbicides	-15.96	-16.31	-16.19	1.43
(4E,8E)-sphinga-4,8-dienine	Other Pathways	Sphingadienine	-0.24	-3.69	-1.3	-3.5
1-monopalmitoylethylene glycol	Other Pathways	Compounds	-0.42	-4.66	-1.34	-5.33
gondoate	Other Pathways	Long-Chain-234-Saturated-Fatty-Acids // Monounsaturated-fatty-acids // Omega-9-Fatty-Acids	1.41	-2.55	-1.66	-19.73
sphinganine (C20)	Other Pathways	D-erythro-Shinganines	-1.4	-1.38	-0.75	-0.78
2-S-cysteinyldopa	Other Pathways	Quinones	18.18			
(8E)-4-hydroxy-sphing-8-enine	Other Pathways	CPD-17589	-0.45	-3.57	-1.36	-5.5
p-mercuribenzoate	Other Pathways	Compounds	0.32	1.21	0.44	0.65
coronatine	Other Pathways	Compounds	0.2	-1.25	0.44	-2.52
S-sulfanylglutathione (GSSH)	Other Pathways	Perthiols // S-Substituted-Glutathione	19.39	5.81	11.86	8.73
F-dUMP	Other Pathways	Compounds	1.41	-1.45	0.79	0.63
(S)-corexime	Other Pathways	ALKALOID	0.26	-0.09	0.03	19.25

diphosphoglycolyl proline	Other Pathways	Compounds	-0.97	-1.27	0.43	-0.54
2'-deoxyadenosine 3'-monophosphate	Other Pathways	Purine-deoxyribonucleotides // Deoxy-Ribonucleoside-3P	0.48	1.83	0.2	-1.44
gibberellin A7	Other Pathways	Gibberellins	-1.65	0.22	2.38	-3.35
3-dehydrosphinganine (C20)	Other Pathways	CPD-22221	-0.41	-1.34		-1.19
1,3-dioctanoyl-1,2,3-butanetriol	Other Pathways	Compounds	0.26	-0.09	0.03	19.68
1,2-dioctanoyl-1,2,4-butanetriol	Other Pathways	Compounds	-0.46	-1.75	-0.54	-2.78
2'-deoxyguanosine 3'-monophosphate	Other Pathways	Purine-deoxyribonucleotides // Deoxy-Ribonucleoside-3P	0.01	2.59	-1.82	2.65
4-amino-2-trifluoromethyl 5-hydroxymethylpyrimidine pyrophosphate	Other Pathways	Compounds	17.36	17.13	17.91	-0.18
9-[6(RS)-C-carboxamido-5,6,7-trideoxy-Œ≤-D-ribo-octofuranosyl]-9H-purin-6-amine	Other Pathways	Compounds	0.49	-1.16	-0.21	1.17
tetraphosphate	Other Pathways	Polyphosphates	-0.35	-0.82	-0.88	-22.35
guazatine	Other Pathways	Compounds	18.17	-0.09	0.03	-0.18
guanosine 3'-monophosphate (3'-GMP)	Other Pathways	3-Phosphomonucleotides // All-Nucleosides	18.65	18.22	17.71	18.41
1,2-dioctanoyl-1-amino-2,3-propanediol	Other Pathways	Compounds	0.07	-0.98	-0.7	-1.04
2-(3-benzoylphenyl)-3,5,7-trihydroxychromen-4-one	Other Pathways	Flavonol-Derivatives	-0.6	-1.52	-0.84	-2
1,2-dioctanoyl-3-methyl-1,2,3-butanetriol	Other Pathways	Compounds	-0.08	2.43	-0.04	1.45
gibberellin A46	Other Pathways	Gibberellins	2.24	0.87	2.73	1.39
2,3-dioctanoylglyceramide	Other Pathways	Compounds	1.37	-2.06	-0.01	-2.2
2'-deoxyuridine 5'-Œ±,Œ≤-imido-diphosphate	Other Pathways	Compounds	0.26	17.57	0.03	18.53
gibberellin A28	Other Pathways	Gibberellins	-0.37	-1.23	-1.06	-1.45
N2-hydroxyguanosine 5'-monophosphate	Other Pathways	Compounds	1.34	-0.02	0.21	-18.98

3,5-dihydroxy-6,7-didehydro-12'-apo-Œ≤-caroten-12'-al	Other Pathways	12-apo-Carotenals	0.63	-0.79	1.01	-0.79
1-palmitoleylglycerol 3-phosphate	Other Pathways	ACYL-SN-GLYCEROL-3P	0.26	-0.09	0.03	20.24
piericidin A	Other Pathways	Antibiotics	0.85	-19.93	1.07	-20.02
5'-(p-nitrophenyl)thioadenosine	Other Pathways	C-nitro-compounds	1.66	-19.38	0.36	-19.37
tetrahydrothiamine diphosphate	Other Pathways	ORGANOSULFUR	17.59	17.46	0.03	18.1
puromycin	Other Pathways	Peptidyl-nucleosides	0.71	1.07	1.18	1.31
scutellarin	Other Pathways	Flavones	1.93	0.75	0.52	-4.81
4-methylumbelliferyl-Œ≤-D-cellobioside	Other Pathways	Compounds	5.97	-11.63	6.97	1
N10-(bromoacetyl)-5,8-dideazafolate	Other Pathways	Bromoaromtic-Compounds	0.4	-16.94	0.94	-17.03
CDP-Œ±-D-glucose	Other Pathways	NDP-alpha-D-glucoses // CDP-SUGARS	-16.81	-0.42	-0.24	-0.79
uridine-5'-diphosphate bromoacetol	Other Pathways	Bromoaromtic-Compounds // Pyrimidines	-0.08	-0.28	-0.23	-0.48
all-trans-phytoene	Other Pathways	carotenoids	17.92	20.79	0.03	20.62
bisorganyltrisulfane	Other Pathways	Compounds	17.44	-0.09	0.03	-0.18
5,10-methylenetetrahydropteroyl tri-L-glutamate	Other Pathways	METHYLENE-THF-GLU-N	1.26	-17.53	-17.43	-17.62
plastoquinone-9	Other Pathways	PLASTOQUINONE	0.23	-0.39	0.04	-1.07
pelargonidin-3,5-diglucoside-5-O-caffeoylglucoside	Other Pathways	Compounds	0.46	-0.21	0.03	-0.89
1,2-dipalmitoyl-phosphatidylglycerol-phosphate	Other Pathways	L-1-PHOSPHATIDYL-GLYCEROL-P	16.97	19.65	17.65	20.14
maltopentaose	Other Pathways	Linear-Malto-Oligosaccharides	0.23	-17.6	0.3	-17.54
ubiquinone-10	Other Pathways	Ubiquinones	-5.75	-18.14	0.65	-12.05
feruloylacetyl-CoA	Secondary Metabolite Biosynthesis	All-Coas	-16.28	1.1	0.59	0.76
(9Z,12Z,15Z)-hexadeca-9,12,15-trienoyl-CoA	Secondary Metabolite Biosynthesis	Long-Chain-Acyl-CoAs	-0.61	-0.57	-0.14	-17.88
N-methylaminobutanal	Secondary Metabolite Biosynthesis	Compounds	18.73	6.11	18.18	12.36
(2S)-methylbutanenitrile	Secondary Metabolite Biosynthesis	Nitriles	0.22	18.15		
des-methyl avenacin	Secondary	Triterpenes	0.26	-0.09	16.99	-0.18

A-1	Metabolite Biosynthesis					
2-hydroxy-2-methylpropanenitrile	Secondary Metabolite Biosynthesis	Cyanohydrins // Aliphatic-S-hydroxynitriles	-19.74	-13.76	-6.64	-13.34
allylsulfenate	Secondary Metabolite Biosynthesis	Alkyl-Sulfenates // ORGANOSULFUR	6.19	7.16	6.95	6.35
(Z)-2-methylpropanal-oxime	Secondary Metabolite Biosynthesis	2-methylpropanaloxime	-1.06	-1.47	-0.1	-2.3
phenylacetaldehyde	Secondary Metabolite Biosynthesis	Aryl-Aldehyde	0.74	-1.86	-0.43	-3.08
2-phenylethylamine	Secondary Metabolite Biosynthesis	Aralkylamines	-0.88	-2.53	-2.54	-2.86
tropinone	Secondary Metabolite Biosynthesis	Compounds	-0.85	-2.55	-2.44	-2.92
dimethylsulfoniopropanoate-amine	Secondary Metabolite Biosynthesis	ORGANOSULFUR	0.98	-0.08	-0.39	-1.03
3,6-dihydronicotinate	Secondary Metabolite Biosynthesis	Vitamins	-0.52	-0.82	-0.25	-2.19
phloroglucinol	Secondary Metabolite Biosynthesis	Compounds	-21.73	1.6	-0.1	1.59
indolin-2-one	Secondary Metabolite Biosynthesis	Indole-Derivatives	0.47	3.36	0.31	3.11
indoxyl	Secondary Metabolite Biosynthesis	Indole-Derivatives	18.17			
tyramine	Secondary Metabolite Biosynthesis	Aralkylamines	0.75	-1.88	-0.46	-3.08
5-oxooctanal	Secondary Metabolite Biosynthesis	Compounds	-2.28	-1.57	1.61	1.6
conhydrine	Secondary Metabolite Biosynthesis	ALKALOID	-0.18	-18.04	-0.06	-18.14
Œ≥-coniceine	Secondary Metabolite Biosynthesis	ALKALOID	-0.03	-17.89	-17.76	-17.98
nicotinate	Secondary Metabolite Biosynthesis	B-vitamins	0.26	-0.09	0.03	18.83
HBOA	Secondary Metabolite Biosynthesis	Benzoxazinoids	0.26	20.13	0.03	18.96
L-phenylalanine	Secondary Metabolite Biosynthesis	Amino-Acids-20 // Aromatic-L-Amino-Acids // Non-polar-amino-acids //	0.26	-0.09	0.03	20.17

		Phenylpropanoids				
4-coumaraldehyde	Secondary Metabolite Biosynthesis	Phenylpropanoids	0.62	-3.03	-0.51	-3.37
(+)-carvone	Secondary Metabolite Biosynthesis	Carvones	0.6	-0.84	-0.14	-0.66
nornicotine	Secondary Metabolite Biosynthesis	Compounds	-0.07	-1.37	-0.53	-2.42
xanthine	Secondary Metabolite Biosynthesis	Compounds	0.27	-0.89	0.08	-1.66
(S)-4-hydroxymandelonitrile	Secondary Metabolite Biosynthesis	Aromatic-S-hydroxynitriles	0.22	20.58		20.61
(3E)-4,8-dimethylnona-1,3,7-triene	Secondary Metabolite Biosynthesis	Homoterpenoids	0.9	-0.49	0.18	0.15
alliin	Secondary Metabolite Biosynthesis	S-Alkyl-L-Cysteine-S-Oxides // ORGANOSULFUR	0.03	0.03	0.03	21.8
methylcinnamate	Secondary Metabolite Biosynthesis	Aromatics	14.68	14.3	0.04	20.65
tryptamine	Secondary Metabolite Biosynthesis	Indole-Derivatives	-1.62	-1.38	-0.2	-1.35
4-hydroxyphenylbutan-2-one	Secondary Metabolite Biosynthesis	POLYKETIDE	0.26	-0.09	0.03	21.24
(6E)-8-oxogeranial	Secondary Metabolite Biosynthesis	Monoterpenals	0.26	19.83	0.03	21.06
3-methoxytyramine	Secondary Metabolite Biosynthesis	Guaiacols	-0.63	1.26	0.24	1.62
(E)-7-(methyltsulfanyl)heptanal oxime	Secondary Metabolite Biosynthesis	CPD-19472	-1.39	-1.1	0.52	-2.31
(Z)-3-(2,4-dihydroxyphenyl)-prop-2-enoate	Secondary Metabolite Biosynthesis	Phenylpropanoids // Aromatic-Acid	0.26	17.27	0.03	18.46
2,4-dihydroxycinnamate	Secondary Metabolite Biosynthesis	Compounds	0.94	0.71	-16.53	1.9
dihydroconiferyl aldehyde	Secondary Metabolite Biosynthesis	Compounds	0.02	1.74	0.41	3.03
3,6-dihydronicotine	Secondary Metabolite Biosynthesis	ALKALOID	-0.68	-0.25	-0.63	-0.38
Œ≤-fenchene	Secondary Metabolite Biosynthesis	Compounds	-13.07	-13.07	-13.07	-13.07
4-(1-methyl-2-	Secondary	Compounds	0.97	-2.43	-0.47	-2.78

pyrrolidinyl)-3-oxobutanoate	Metabolite Biosynthesis					
2-[(2'-methylsulfanyl)propyl]maleate	Secondary Metabolite Biosynthesis	CPD-19484	-0.75	-1.94	0.12	-0.95
N-hydroxy-L-dihomomethionine	Secondary Metabolite Biosynthesis	N-hydroxy-n-homo-methionine	-0.48	0.29	0.44	-0.64
caffeine	Secondary Metabolite Biosynthesis	Compounds	-0.78	0.82	0.36	0.92
N,N-dihydroxy-L-phenylalanine	Secondary Metabolite Biosynthesis	N-hydroxylated-Amino-Acids	0.3	-1.83	0.12	
leucodopachrome	Secondary Metabolite Biosynthesis	ALKALOID // Indole-Derivatives	-11.05	-5.92	-11.27	6.47
3-carboxy-6-(methylsulfanyl)-2-oxohexanoate	Secondary Metabolite Biosynthesis	CPD-21521	-1.25	-1.29	-0.01	-2.8
(E)-œâ-(methylsulfanyl)heptyl-thiohydroximate	Secondary Metabolite Biosynthesis	n-methylthioalkylhydroximate	0.26	19.15	0.03	19.67
3-oxo-dodecanoate	Secondary Metabolite Biosynthesis	CPD-12642	-0.2	-1.42	-0.25	-2.45
bergapten	Secondary Metabolite Biosynthesis	Furanocoumarins	0.26	-0.09	0.03	21.27
3-deoxycapsidiol	Secondary Metabolite Biosynthesis	Sesquiterpenes	0.26	-0.09	0.03	19.98
[(4S)-4-(5,5-dimethylcyclohex-1-en-1-yl)-cyclohex-1-en-1-yl] methanol	Secondary Metabolite Biosynthesis	Sesquiterpenes	7.56	7.19	7.4	7.8
3-[(3'-methylsulfanyl)propyl]malate	Secondary Metabolite Biosynthesis	CPD-19483	0.77	1.55	0.74	2.33
(3E,7E)-4,8,12-trimethyltrideca-1,3,7,11-tetraene (TMTT)	Secondary Metabolite Biosynthesis	Homoterpenoids	0.4	-1.32	-0.09	-0.2
coniferyl acetate	Secondary Metabolite Biosynthesis	Coniferyl-Esters // Secondary-Metabolites	-0.61	-0.36	0.9	0.72
pinosylvin monomethylether	Secondary Metabolite Biosynthesis	Compounds	7.14	-6.78	-6.66	15.4
(R)-5-phosphomevalonate	Secondary Metabolite Biosynthesis	Compounds	0.26	-0.09	0.03	17.19
(+)-heliannuol K	Secondary Metabolite Biosynthesis	Sesquiterpenes	0.22	-0.13	9.83	19.65
(-)-patchoulol	Secondary Metabolite	Sesquiterpenes	1.1	-0.19	-0.27	-0.24

	Biosynthesis					
petivericin	Secondary Metabolite Biosynthesis	CPD-17425	-0.38	-1.86	-0.45	-2.5
heliannuol C	Secondary Metabolite Biosynthesis	Sesquiterpenes	1.01	2.1	-0.08	0.13
2-[(2'-methylsulfanyl)heptyl]maleate	Secondary Metabolite Biosynthesis	CPD-19484	-1.51	-0.61	-1.65	-15.15
2-[(6'-methylsulfanyl)hexyl]malate	Secondary Metabolite Biosynthesis	CPD-19482	20.33			19.03
D-mannitol 1-phosphate	Secondary Metabolite Biosynthesis	Alditol-phosphates	0.26	-0.09	0.03	19.59
2-carboxy-D-arabinitol 1-phosphate	Secondary Metabolite Biosynthesis	Compounds	0.26	17.86	0.03	18.39
4'-O-methylnorbelladine	Secondary Metabolite Biosynthesis	Compounds	0.25	14.15	0.07	7.13
2'-O-methylisoliquiritigenin	Secondary Metabolite Biosynthesis	Compounds	-1.7	0.29	0.28	-0.95
(3R,4R)-7,2',4'-trihydroxyisoflavanol	Secondary Metabolite Biosynthesis	4R-4-2prime-dihydroxyisoflavans	0.26	-0.09	0.03	19
7-hydroxy-4'5'-dimethoxyisoflavone	Secondary Metabolite Biosynthesis	Isoflavones	0.65	-0.81	0.19	-1.15
3-[(7'-methylsulfanyl)heptyl]malate	Secondary Metabolite Biosynthesis	CPD-19483	0.71	-0.58	0.27	-0.96
2-phenylethyl Œ≤-D-glucopyranoside	Secondary Metabolite Biosynthesis	Compounds	-2.67	-2.53	-1.61	-0.98
xanthosine	Secondary Metabolite Biosynthesis	Purine-Ribonucleosides	0.15	-0.92	-0.11	-1.26
11-hydroxyvittatine	Secondary Metabolite Biosynthesis	Compounds	-21.86	0.4	0.37	-1.16
lycorine	Secondary Metabolite Biosynthesis	Compounds	2.47	0.4	0.37	-1.16
shikonin	Secondary Metabolite Biosynthesis	Quinones // Secondary-Metabolites	0.54	2.12	0.96	2.68
ponciretin	Secondary Metabolite Biosynthesis	4-Methoxyflavanones	-1.85	-3.58	-2.51	-3.7
manoyl oxide	Secondary Metabolite Biosynthesis	Diterpenes	0.46	-0.76	-0.72	-3.66
(-)-epicatechin	Secondary	CPD-10414	1.15	-1.72	0.77	-1.74

	Metabolite Biosynthesis					
(2R,3S,4S)-leucopelargonidin	Secondary Metabolite Biosynthesis	CPD-11512	18.03			19.27
salicylate β-D-glucose ester	Secondary Metabolite Biosynthesis	Glycosyl-Esters // Aromatics	-0.96	-1.04	0.11	1.41
(S)-N-methylcoclaurine	Secondary Metabolite Biosynthesis	Compounds	21.74	-0.05		21.02
3''-deamino-3''-oxonicotianamine	Secondary Metabolite Biosynthesis	Compounds	0.42	-0.94	0.09	-1.38
plaunotol	Secondary Metabolite Biosynthesis	Isoprenoid-Derivatives	-0.93	-0.15	-0.95	0.08
(R)-mevalonate diphosphate	Secondary Metabolite Biosynthesis	Compounds	0.26	16.78	0.03	17.92
geranylgeraniol	Secondary Metabolite Biosynthesis	Methyl-Branched-Lipids // Prenols	0.26	-0.09	0.03	17.92
cinnamoyl-Œ≤-D-glucoside	Secondary Metabolite Biosynthesis	Beta-D-glucosides	-0.33	-5.92	-5.96	-7.39
chrysanthemyl diphosphate	Secondary Metabolite Biosynthesis	Compounds	-0.84	1.95	0.66	1.54
dhurrin	Secondary Metabolite Biosynthesis	Nitriles	0.19	-0.93	-0.2	-1.12
(Z)-1-(L-cysteinyglycin-S-yl)-N-hydroxy-2-phenylethan-1-imine	Secondary Metabolite Biosynthesis	CPD-6262	-18.26	1.73	0.37	1.35
kauralexin A3	Secondary Metabolite Biosynthesis	Kauralexins	1.47	-0.1	2.31	-1.23
(S)-3'-hydroxy-N-methylcoclaurine	Secondary Metabolite Biosynthesis	Compounds	-0.26	-0.58	0.06	-1.64
ent-cassa-12,15-diene-2Œ≤,3Œ≤-diol-11-one	Secondary Metabolite Biosynthesis	Diterpenes	-0.71	-2.05	-1.42	-3.32
peonidin	Secondary Metabolite Biosynthesis	Anthocyanidins	-0.02	-0.17	-0.02	13.05
(2R,3S,4S)-leucodelphinidin	Secondary Metabolite Biosynthesis	CPD-11512	0.33	-1.2	-0.16	-1.61
quinine	Secondary Metabolite Biosynthesis	Quinoline-MIAs	10.33	-2.35	10.09	12.68
(S)-cheilanthifoline	Secondary Metabolite Biosynthesis	Compounds	20.38			

hyperxanthone E	Secondary Metabolite Biosynthesis	Secondary-Metabolites // Aromatics	-0.19	-0.87	0.38	-0.92
taxa-4(20),11-dien-5-Œ±-yl acetate	Secondary Metabolite Biosynthesis	Compounds	-4.62	-6.04	-5.17	-6.04
3,7-dimethylquercetin	Secondary Metabolite Biosynthesis	Flavonol-Derivatives	0.26	19.64	0.03	19.13
4-coumaroyl-4'-hydroxyphenyllactate	Secondary Metabolite Biosynthesis	Compounds	-0.1		18.9	
robustaquinone A	Secondary Metabolite Biosynthesis	Hydroxyanthraquinones	20.85	20.72	0.03	21.21
9-demethylmunduserone	Secondary Metabolite Biosynthesis	Compounds	0.26	-0.09	0.03	18.5
kauralexin A2	Secondary Metabolite Biosynthesis	Kauralexins	0.26	-0.09	0.03	20.83
demethoxycurcumin	Secondary Metabolite Biosynthesis	Secondary-Metabolites	0.26	21.92	0.03	13.94
columbamine	Secondary Metabolite Biosynthesis	Compounds	23.4		19.42	
glyceollin II	Secondary Metabolite Biosynthesis	Glyceollins	14.61	15.63	-6.29	7.64
sinapoyl-(S)-malate	Secondary Metabolite Biosynthesis	Compounds	0.77	-0.29	-0.04	-1.64
3,7-dimethylmyricetin	Secondary Metabolite Biosynthesis	Flavonol-Derivatives	-0.18	-1.56	0.46	-3.56
13-[(E)-2-methylcrotonoyl]oxylupanine	Secondary Metabolite Biosynthesis	Compounds	0.39	-0.83	0.37	-1.43
polyneuridine aldehyde	Secondary Metabolite Biosynthesis	Compounds	0.26	-0.09	0.03	22.37
hinokinin	Secondary Metabolite Biosynthesis	LIGNAN	1.74	3.4	-0.83	3.76
carnosate	Secondary Metabolite Biosynthesis	Diterpenes	0.26	-0.09	0.03	20.41
scopolin	Secondary Metabolite Biosynthesis	Compounds	1.23	0.81	-0.77	-0.34
2,3-dehydrokievitone	Secondary Metabolite Biosynthesis	Compounds	0.33	19.58	0.27	12.74
gluconapin	Secondary Metabolite Biosynthesis	alkenyl-glucosinolate	-0.51	-19.05	-0.56	-19.15

D-galactosylononitol	Secondary Metabolite Biosynthesis	Methyl-Inositols // Galactosyl-Inositols	-18.75	0.95	1.87	0.61
sinigrin	Secondary Metabolite Biosynthesis	alkenyl-glucosinolate	-0.88	-2.28	-0.75	-2.44
rosmarinate	Secondary Metabolite Biosynthesis	Compounds	-17.79	3.21	-18.01	3.06
gibberellin A37	Secondary Metabolite Biosynthesis	Gibberellins	0.26	-0.09	0.03	19.73
7-(methylsulfanyl)heptyl-desulfoglucosinolate	Secondary Metabolite Biosynthesis	Desulfoaliphaticglucosinolates	1.37	-2.02	0.38	-2.96
dopaxanthin quinone	Secondary Metabolite Biosynthesis	ALKALOID	-1.43	-18.05	-1.37	-6.44
2'-hydroxy 3,6,7,3',4'-pentamethylquercetagetin	Secondary Metabolite Biosynthesis	Flavonol-Derivatives	1.64	-0.04	0.6	-0.04
dopaxanthin	Secondary Metabolite Biosynthesis	ALKALOID	1.59	-19.35	0.39	-19.45
O-sinapoylglucarate	Secondary Metabolite Biosynthesis	Compounds	1.23	-16.93	-0.12	-4.56
(-)-yatein	Secondary Metabolite Biosynthesis	LIGNAN	0.95	-3.03	0.27	-3.97
glucoraphanin	Secondary Metabolite Biosynthesis	omega-methylsulfinylalkylglucosinolate	-0.01	-1.72	-0.15	-1.82
oleanolic aldehyde	Secondary Metabolite Biosynthesis	Triterpenes	1.63	1.67	0.9	0.43
baicalin	Secondary Metabolite Biosynthesis	Flavones	-1.19	0.3	0.8	-0.7
(-)-maackiain-3-O-glucoside	Secondary Metabolite Biosynthesis	Compounds	-1.19	-0.83	0.59	-0.02
<i>N</i>-β-D-glucosyl-(indol-3-yl)acetyl-L-aspartate	Secondary Metabolite Biosynthesis	Indole-Derivatives // Modified-Amino-Acids	1.58	1.18	0.3	1.56
hederagenin	Secondary Metabolite Biosynthesis	Triterpenes	1.01	-0.15	-0.46	-0.05
isorhamnetin 3-O-glucoside	Secondary Metabolite Biosynthesis	Flavonol-3-O-Glycosides	1.11	-6.73	-0.96	-11.95
4-methoxyglucobrassicin	Secondary Metabolite Biosynthesis	Indolic-glucosinolates	-0.87	0.49	0.81	2.15

quercetin-3-glucoside	Secondary Metabolite Biosynthesis	Flavonol-3-O-B-D-Glucosides	0.26	18.97	0.03	18.82
quercetin 3-galactoside	Secondary Metabolite Biosynthesis	Flavonol-3-O-Glycosides	-1.29	-1.03	0.7	-1.17
glucomalcommin	Secondary Metabolite Biosynthesis	Aliphatic-glucosinolates	0.22	20.47	17.25	20.46
luteolin 7-O-Œ≤-D-glucuronide	Secondary Metabolite Biosynthesis	Compounds	0.33	12.02	0.1	11.99
8-(methylsulfanyl)octyl-glucosinolate	Secondary Metabolite Biosynthesis	Omega-methylthio-alkyl-glucosinolates	-0.3	2.37	-0.28	-0.69
patulitrin	Secondary Metabolite Biosynthesis	Flavonol-Derivatives	1.91	-16.74	0.26	-16.83
3-(O-β-glucosyl)-(2-oxindol-3-yl)acetyl-L-aspartate	Secondary Metabolite Biosynthesis	Indole-Derivatives // Modified-Amino-Acids	0.97	2.21	0.3	1.85
8-(methylsulfinyl)octyl-glucosinolate	Secondary Metabolite Biosynthesis	omega-methylsulfinylalkylglucosinolate	6.79	18.31	18.06	18.46
2-benzoyloxy-3-butenylglucosinolate	Secondary Metabolite Biosynthesis	Compounds	-17.58	0.63	-0.33	0.14
4-benzoyloxybutylglucosinolate	Secondary Metabolite Biosynthesis	Compounds	6.23	-11.51	-11.4	1.03
hypericin	Secondary Metabolite Biosynthesis	Dyes	0.26	17.1	17.53	16.49
malonylgenistin	Secondary Metabolite Biosynthesis	Compounds	19.96	-0.09	0.03	-0.18
malonyldaidzin	Secondary Metabolite Biosynthesis	Compounds	-16.97	-0.09	0.13	0.33
eupatolitin 3-glucoside	Secondary Metabolite Biosynthesis	Flavonol-Derivatives	1.37	0.4	-0.24	-6.5
cyclo-dopa glucuronosyl glucoside	Secondary Metabolite Biosynthesis	ALKALOID	0.26	-0.09	16.53	-0.18
curcumin monoglucoside	Secondary Metabolite Biosynthesis	POLYKETIDE // Phenols // Phenylpropanoids	1.11	-0.13	0.16	-0.78
raucaffricine	Secondary Metabolite Biosynthesis	Ajmalan-MIAs	0.94	1.36	1.31	1.61
cyanidin 3-O-(6"-O-malonyl)-Œ≤-glucoside	Secondary Metabolite Biosynthesis	Anthocyanidin-malonyl-beta-D-glucosides	0.26	19.32	0.03	-0.03
3,7,3',4'-tetramethylquerceti	Secondary Metabolite	Flavonol-Derivatives	-0.11	-4.74	2.24	-4.31

n 2'-O-Œ≤-D-glucoside	Biosynthesis					
pelargonidin 3-O-(6''-O-malyl-Œ≤-D-glucoside)	Secondary Metabolite Biosynthesis	Anthocyanins	0.28	-1.73	-0.99	0.34
cyanidin 3-O-(6''-O-malyl-Œ≤-D-glucoside)	Secondary Metabolite Biosynthesis	Anthocyanidin-malonyl-beta-D-glucosides	-0.25	-17.39	0.04	-17.49
9,9'-di-cis-Œ∂-carotene	Secondary Metabolite Biosynthesis	Carotenes	0.53	-0.27	-0.16	-0.27
pelargonidin 3-O-Œ≤-D-sambubioside	Secondary Metabolite Biosynthesis	Anthocyanins	1.29	0.14	-0.53	-0.09
canthaxanthin	Secondary Metabolite Biosynthesis	Ketocarotenoids	0.41	-1.39	-0.56	-0.82
isovitexin-7-O-xyloside	Secondary Metabolite Biosynthesis	Flavones	0.26	18.72	0.03	18.99
15-cis-phytoene	Secondary Metabolite Biosynthesis	carotenoids	0.26	20.79	0.03	20.62
gomphrenin I	Secondary Metabolite Biosynthesis	ALKALOID	0.22	18.13	0.03	16.63
maysin	Secondary Metabolite Biosynthesis	Flavones	-1.12	-1.83	-1.2	-19
vitexin 2''-O-Œ≤-L-rhamnoside	Secondary Metabolite Biosynthesis	Compounds	1.79	0.12		-5.82
isovitexin 2''-O-Œ≤-D-glucoside	Secondary Metabolite Biosynthesis	Compounds	-19.21	-19.21	-1.11	-19.29
apigenin 7-O-[Œ≤-D-apiosyl-(1,Üí2)-Œ≤-D-glucoside]	Secondary Metabolite Biosynthesis	Compounds	1.74	1.26	0.08	1.54
4-sinapoyloxybutylglucosinolate	Secondary Metabolite Biosynthesis	Compounds	-10.71	-10.77	-10.94	8.26
cyanidin 3-O-(Œ≤-D-xylosyl-(1,Üí2)-Œ≤-D-galactoside)	Secondary Metabolite Biosynthesis	Anthocyanins	17.8	-0.09	0.03	-0.19
isorhamnetin 3-O-(3''-O-p-coumaroyl)-glucoside	Secondary Metabolite Biosynthesis	Flavonol-Derivatives	0.08	-1.59	-0.44	-2.63
quercetin 3-O-(4''-O-p-coumaroyl)-glucoside	Secondary Metabolite Biosynthesis	Flavonol-Derivatives	0.68	-0.22	0.22	-1.05
quercetin 3, 3', 4', 7-tetrasulfate	Secondary Metabolite Biosynthesis	Flavonol-Derivatives	0.19	11.58	17.05	10.78
quercetin 3,7-O-diglucoside	Secondary Metabolite Biosynthesis	Flavonol-Derivatives	5.58	-5.66	7.18	-11.26

<i>N</i>-[β-glucosyl-(1,4)-β-glucosyl]-(oxindol-3-yl)acetyl-aspartate	Secondary Metabolite Biosynthesis	Indole-Derivatives // Modified-Amino-Acids	-19.27	-19.54	-19.27	-7.32
dalnigrein 7-O-Œ≤-D-apiofuranosyl-(1-6)-Œ≤-D-glucopyranoside	Secondary Metabolite Biosynthesis	Isoflavones	17.36	-0.08		-0.03
rubusoside	Secondary Metabolite Biosynthesis	Diterpenes	0.32	-2.12	1.21	
16-alpha-hydroxygypsogenate-28-beta-D-glucoside	Secondary Metabolite Biosynthesis	Triterpenes	-0.02	-2.38	-0.34	-21.62
N-debenzoyl-(3'-RS)-2'-deoxytaxol	Secondary Metabolite Biosynthesis	ALKALOID	6.95	6.07	6.95	-0.61
dihydroxyferuloyl-sinapoyl spermidine	Secondary Metabolite Biosynthesis	Compounds	17.61	-0.09	0.03	-0.18
N-debenzoyl-(3'-RS)-taxol	Secondary Metabolite Biosynthesis	ALKALOID	17.31	16.98	0.03	18.06
quercetin 3-O-(3',6''-O-di-p-coumaroyl)-glucoside	Secondary Metabolite Biosynthesis	Flavonol-Derivatives	0.6	-19.2		-1.01
kaempferol 3-O-(4'',6''-O-di-p-coumaroyl)-glucoside	Secondary Metabolite Biosynthesis	Flavonol-Derivatives	0.49	-0.2	0.06	-0.89
cyanidin 3-O-(6-O-Œ≤-D-glucosyl-2-O-Œ≤-D-xylosyl-Œ≤-D-galactoside)	Secondary Metabolite Biosynthesis	Anthocyanins	0.58	-0.15	0.09	-0.9
(20S)-ginsenoside Rg3	Secondary Metabolite Biosynthesis	3GlcGlc-Protopanaxadiol-ginsenosides	1.12	4.44	0.63	5.08
oleanolate 3-beta-D-glucuronoside-(3,1)-galactoside	Secondary Metabolite Biosynthesis	Triterpenes	18.31	18.16	17.42	19.13
monodeglucosyl des-acyl avenacin A	Secondary Metabolite Biosynthesis	Triterpenes	16.41	14.14	16.91	5.76
Œ≤-D-gentiobiosyl Œ≤-D-glucosyl crocetin	Secondary Metabolite Biosynthesis	Beta-D-glucosides // Glycosyl-Esters	18.07	-0.1	0.03	10.83
vincristine	Secondary Metabolite Biosynthesis	Organic-heteroptetracyclic-compounds	19.21	-0.1	0.04	13.52
curcumin 4'-O-Œ≤-D-gentiotrioside	Secondary Metabolite Biosynthesis	POLYKETIDE // Phenols // Phenylpropanoids	-0.08	-18.21	-1.19	-18.32
ternatin C5	Secondary Metabolite Biosynthesis	Anthocyanins	-16.43	-16.78	-0.09	-16.87

cyanidin O-O-[6-O-(6-O-4-hydroxycinnamoyl-Œ≤-D-glucosyl)-2-O-Œ≤-D-xylosyl-Œ≤-D-galactoside]	Secondary Metabolite Biosynthesis	Anthocyanins	0.23	-18.02	-0.08	-18.12
kaempferol 3-O-[6-(4-coumaroyl)-Œ≤-D-glucosyl-(1->2)-glucosyl-(1->2)-Œ≤-D-glucoside	Secondary Metabolite Biosynthesis	Flavonol-Derivatives	-0.35	-0.05	0.27	-0.67
(2R)-2-hydroxy-2-methylbutanenitrile	Secondary Metabolite Degradation	2-Hydroxy-2-methylbutyronitriles // Aliphatic-R-hydroxynitriles	-20.41	0.88	0.01	1.52
indole-3-carbinol	Secondary Metabolite Degradation	Indole-Phytoalexins	-1.73	-1.62	-0.73	-0.33
benzylisothiocyanate	Secondary Metabolite Degradation	ISOTHIOCYANATES	-0.7	-6.65	-0.14	0.04
benzylthiocyanate	Secondary Metabolite Degradation	Compounds	0.26	-0.09	0.03	18.36
pseudoionone	Secondary Metabolite Degradation	Methylketones	0.26	-0.09	0.03	18.78
2-benzyl-thiohydroximate-O-sulfate	Secondary Metabolite Degradation	THIOHYDROXIMATE-O-SULFATES	0.26	18.21	0.03	21.06
7'-hydroxyabscisate	Secondary Metabolite Degradation	Abscisic-Acid-Derivative	0.26	23.89	0.03	22.81
primeverose	Secondary Metabolite Degradation	Disaccharides	-7.33	14.64	-7.65	14.66
(glutathion-S-yl)-4-methoxy-3-indolylmethylisothiocyanate	Secondary Metabolite Degradation	Indole-Phytoalexins // S-Substituted-Glutathione	-18.52	-1.03	0.82	0.74
