# Supplementary material for: Metabolomics and Physiological Insights into the Ability of Exogenously Applied Chlorogenic Acid and Hesperidin to Modulate Salt Stress in Lettuce Distinctively
Source: Molecules. 2021 Oct 18;26(20):6291. doi: 10.3390/molecules26206291 (PMC8537907; doi:10.3390/molecules26206291)
Supplement: Supplementary file 1 [file molecules-26-06291-s001.zip › Table S4.pdf]

Table S4. List of most discriminant metabolites having a variable importance in projection (VIP) score greater than 1.3, extrapolated from the orthogonal projections to latent structures discriminant analysis (OPLS-DA) model. For each compound were reported the compound ontology, VIP score, and standard error.

Compound name	Ontology - parents of class	VIP score	Standard error
<i>N</i>⁶-(Δ²-isopentenyl)-adenosine 5'-diphosphate	Compounds	1,46038	0,367011
1-(5-phospho-β-D-ribosyl)-AMP	Modified-Nucleosides	1,45127	0,616705
sphinganine (C20)	D-erythro-Shinganines	1,44285	0,357339
8-hydroxy-dATP	Modified-Nucleotides	1,44199	0,675212
cyclo-dopa glucuronosyl glucoside	Alkaloids	1,44101	0,705058
dihydroxyferuloyl-sinapoyl spermidine	Compounds	1,43063	0,563142
di-homo-γ-linolenate	Omega-6-Fatty-Acids // Eicosatrienoates	1,42857	0,330283
D-<i>myo</i>-inositol (1,2,3,5,6) pentakisphosphate	D-myo-inositol-pentakisphosphates	1,39764	0,528647
spermine	Aliphatic-Alpha-Omega-Diamines	1,3976	0,527045
(2<i>E</i>,10<i>E</i>,14<i>E</i>,18<i>E</i>)-lycopatetraene	Hydrocarbons // Tetraterpenes	1,39735	0,523227
UDP-3-<i>O</i>-[(3<i>R</i>)-3-hydroxymyristoyl]-<i>N</i>-acetyl-α-D-glucosamine	UDP-sugar	1,39472	0,523865
6-(<i>all-trans</i>-decaprenyl)-2-methoxy-phenol	2-Methoxy-6-polyprenyl-phenols	1,39458	0,522689
CDP-ethanolamine	Modified-Nucleotides // Pyrimidines	1,38856	0,43689
<i>N</i>-(4-aminobenzoyl)-L-glutamate	Compounds	1,38457	0,519525
dihydroisopteroate	Pteroates	1,37921	0,520611
nitecapone	C-nitro-compounds	1,37295	0,526361
hemigossypol	Sesquiterpenes	1,37057	0,490254
1-stearoyl-<i>sn</i>-glycerol	1-Acyl-sn-glycerols	1,36431	0,248894
pelargonidin 3-<i>O</i>-(6"-<i>O</i>-malyl-β-<i>D</i>-glucoside)	Anthocyanins	1,36354	0,518353
3-dehydrosphinganine (C20)	Fatty acids	1,35455	0,301674
2-succinyl-5-enolpyruvoyl-6-hydroxy-3-cyclohexene-1-carboxylate	Compounds	1,35337	0,221114
<i>all trans</i>-decaprenyl-2-methoxy-6-1,4-benzoquinol	2-Methoxy-6-Polyprenyl-14-Benzoquinols	1,35292	0,591587
<i>N</i>-(4-hydroxybenzoyl)-L-glutamate	Monocarboxylic-Acid-Amides	1,35054	0,507803
UDP-2-N,3-O-bis[(3R)-3-hydroxytetradecanoyl]-α-D-glucosamine	UDP-sugar	1,34445	0,446606
isopentenyladenine-7-<i>N</i>-glucoside	Adenine-Cytokinin-Glucosides	1,34436	0,190895
7,8-dihydropteroate	Pteroates	1,34353	0,540559
2-thiouridine 5'-triphosphate	Compounds	1,34278	0,270898

hypericin	Dyes	1,33244	0,266173
isopentenyl adenosine	Nucleosides-Analogues	1,32961	0,237565
CDP-α-D-glucose	NDP-alpha-D-glucoses // CDP-SUGARS	1,32749	0,526932
cyclic ADP-ribose	Nucleosides-Analogues	1,32698	0,533581
1,18-octadecane-diol	Long-chain-alcohols // Diols	1,32516	0,355421
lubimin	Sesquiterpenes	1,32411	0,305829
capsidiol	Sesquiterpenes	1,32387	0,30117
dodecane-1,7-diol	Diols	1,32324	0,278343
soyasaponin I	Soyasaponins // Pentacyclic-triterpenoids	1,32293	0,327683
dopaxanthin quinone	ALKALOID	1,32256	0,385137
guanosine tetraphosphate	Modified-Nucleotides	1,32126	0,275948
lauroyl-CoA	Medium-Chain-234-Saturated-acyl-CoAs	1,31932	0,230541
(22R,23R)-22,23-dihydroxy-campest-4-en-3-one	Brassinosteroids	1,31862	0,346691
<i>N</i>¹-acetylspermine	Aliphatic-N-Acetyl-Diamines	1,31848	0,875642
1-(5-phospho-β-D-ribosyl)-5-[(5-phosphoribosylamino)methylideneamino]imidazole-4-carboxamide	Carboxamides	1,31249	0,554949
3-dehydro-6-hydroxyteasterone	Brassinosteroids	1,31201	0,329677
adenosine 5'-phosphoselenate	Compounds	1,30855	0,314754
3R-hydroxy-lesqueroloyl-CoA	All-Coas	1,30477	0,60211
isorhamnetin 3-<i>O</i>-(6"-<i>O</i>-feruloyl)-glucoside	Flavonol-Derivatives	1,30364	0,234924
1-18:2-2-16:0-digalactosyldiacylglycerol	Fatty acids	1,30254	0,283116
